# Identification and Expression Analysis of the Formin Gene Family in *Neopyropia yezoensis* Under Different Environmental Conditions

**DOI:** 10.3390/genes17091089

**Published:** 2026-09-10

**Authors:** Shengqi Ye, Hongxin Ji, Lianxuan Chen, Jingwen Qi, Haihong Chen

**Affiliations:** Jiangsu Key Laboratory of Marine Bioresources and Environment, Jiangsu Key Laboratory of Marine Biotechnology, Jiangsu Ocean University, Lianyungang 222005, China

**Keywords:** *Neopyropia yezoensis*, Formin gene family, abiotic stress response, qRT-PCR, light, temperature, desiccation and rehydration

## Abstract

**Background/Objectives:** *Neopyropia yezoensis* is an economically important intertidal red alga that frequently experiences fluctuations in temperature, light intensity, and water availability. Formins are key regulators of actin nucleation and cytoskeletal dynamics, but their functions and stress-responsive roles in red algae remain poorly understood. This study aimed to systematically identify and characterize the Formin gene family in *N. yezoensis* and investigate their expression responses to different environmental stresses. **Methods:** Formin family members were identified from the *N. yezoensis* genome using HMMER and BLAST (2.17.0) searches based on the conserved FH2 domain, followed by domain validation. Gene structures, conserved motifs, physicochemical properties, predicted subcellular localization, protein structures, chromosomal distribution, phylogenetic relationships, and cis-acting elements in the upstream regions were analyzed. The expression patterns of the identified Formin genes were further examined by qRT-PCR under different temperature (4, 10, and 24 °C), light intensity (20, 60, and 100 μmol photons m^−2^ s^−1^), and desiccation/rehydration conditions. **Results:** Three Formin genes, designated NpyFormin01–03, were identified in *N. yezoensis*. All three encoded proteins contained the conserved FH2 domain but differed in motif composition, domain architecture, predicted subcellular localization, and structural features. NpyFormin01 contained additional PTEN_C2 and PTP_DSP_cys domains, whereas NpyFormin02 and NpyFormin03 contained only the FH2 domain. Phylogenetic analysis showed that the *N. yezoensis* Formins clustered with Formins from other red algae. Promoter analysis identified multiple predicted cis-acting elements associated with light, temperature, environmental, and phytohormone responses. Expression analysis revealed distinct responses among the three genes under the tested environmental conditions. Notably, NpyFormin01 was significantly upregulated under high-temperature treatment (24 °C), whereas NpyFormin02 and NpyFormin03 showed no statistically significant expression changes under the tested conditions. **Conclusions:** This study provides a systematic characterization of the Formin gene family in *N. yezoensis*. The differences in protein architecture, structural features, promoter cis-acting elements, and environmental-responsive expression patterns suggest potential functional divergence among NpyFormins. In particular, NpyFormin01 represents a potential heat-responsive candidate gene and may contribute to cytoskeletal regulation during environmental stress adaptation in *N. yezoensis*. These findings provide a basis for further investigation of the molecular functions of Formins in red algae.

## 1. Introduction

In natural ecosystems, changes in abiotic factors such as light, temperature, water, and nutrients can synergistically or antagonistically affect the growth, development, and metabolism of photosynthetic organisms [1,2,3,4]. Due to tidal influences, intertidal macroalgae frequently experience fluctuations in environmental factors such as water, temperature, light, and nutrients [5]. To cope with the frequent environmental changes in the intertidal zone, macroalgae have evolved sophisticated cellular response systems over long-term evolution. Dynamic reorganization of the cytoskeleton is considered a key pathway by which eukaryotic cells respond to external stimuli, adjust their morphology, and maintain functional integrity, playing an essential role in the environmental adaptation of plants and algae and ensuring normal cellular physiology and healthy thallus development [6,7,8,9,10].

As one of the earliest diverging multicellular eukaryotes, red algae exhibit remarkable uniqueness in their cytoskeletal molecular regulatory systems. Although actin, a core component of the cytoskeleton, is universally present in red algae, its regulatory network has undergone significant simplification [11,12,13,14]. For example, studies on the extremophilic red alga *Cyanidioschyzon merolae* have revealed that it achieves phototactic movement through a novel actin-driven tentacle structure, the formation of which does not rely on the multiple regulatory factors required for classical pseudopodia, such as the ARP2/3 complex and myosin [11]. Genome-wide searches across multiple red algal species have revealed that genes encoding microfilament polymerization- and nucleation-related proteins—including the ARP2/3 complex, WASP, SCAR, and WAVE—are markedly reduced or absent in red algal genomes [12,13,14]. Notably, although red algae lack many genes that regulate microfilament dynamics in animals and plants, their genomes are relatively enriched in genes encoding the linear microfilament nucleator Formin [12].

Formins are a family of multi-domain actin nucleating factors whose ability to regulate cytoskeletal dynamics by mediating actin polymerization has been demonstrated in various eukaryotic organisms [15,16,17,18]. Many Formin proteins contain three main domains: Formin homology domain 1 (FH1), Formin homology domain 2 (FH2), and Formin homology domain 3 (FH3) [19]. However, domain composition varies among different Formin subfamilies and species; the FH2 domain, which is essential for the actin polymerization activity, is the most conserved and is commonly used for gene family identification [20,21]. In land plants, genome-wide identification of the Formin gene family has been accomplished in multiple species, such as soybean, cotton and potato [22,23,24,25]. Functional studies in *Arabidopsis thaliana*, rice (*Oryza sativa*), and cotton (*Gossypium hirsutum* L.) have demonstrated that Formins play important roles in responses to multiple abiotic stresses, including drought, heavy metal stress, and heat stress [26,27,28]. In contrast, functional investigations of Formins in algae have commenced more recently, yet accumulating evidence suggests their physiological relevance [9,14,29]. Notably, a recent study reported that Formin genes in *Neopyropia yezoensis* exhibit altered expression at the transcriptional level, providing preliminary evidence for their potential involvement in environmental stress adaptation in this red alga [9]. These findings, together with the unique enrichment of Formin-encoding genes in red algal genomes [12], point to Formins as key candidates for mediating cytoskeletal dynamics and stress responses in this lineage.

Given the unique cytoskeletal features of red algae and the fluctuating intertidal environment that some members of this group undergo [30], the Formin family in these organisms warrants systematic study. Nevertheless, systematic investigations of the Formin gene family in this lineage—covering genome-wide identification, structural characterization, and expression profiling under diverse stresses—remain unavailable.

*N. yezoensis* is an economically significant intertidal red alga that naturally experiences frequent and concurrent fluctuations in temperature, light intensity, and water availability [30], stresses that are among the most ecologically relevant for intertidal macroalgae. In the present study, a genome-wide identification and structural characterization of the Formin gene family were performed in *N. yezoensis*, and the expression profiles of identified Formin genes under temperature, light, and desiccation stresses were examined using quantitative real-time PCR (qRT-PCR). Collectively, our findings provide novel insights into the potential roles of Formins in environmental adaptation of *N. yezoensis* and contribute to a broader understanding of stress-responsive mechanisms in red algae.

## 2. Materials and Methods

### 2.1. Material

The experimental material used in this study was the gametophytic thalli of *N. yezoensis* strain LL62. These thalli were obtained by cultivating conchospores released from conchocelis maintained in the Phycology Laboratory of Jiangsu Ocean University [31]. Prior to the experiments, the thalli were pre-cultured in Provasoli’s Enriched Seawater (PES) medium at 10 °C under a 12 h light/12 h dark photoperiod with a light intensity of 60 μmol photons m^−2^ s^−1^ [32].

### 2.2. Gene Family Identification

The genome data of *N. yezoensis* were retrieved from the genomic dataset released by Wang et al. (2020) [33]. The specific assembly version used in this study was WMLA01000000 (GenBank accession WMLA00000000). The reference genome assembly comprised 660 contigs with an N50 of 340.8 kb and was further organized into three chromosome-scale scaffolds, which together accounted for approximately 95% of the assembled genome sequence. The genome size was approximately 108 Mb, and 12,855 protein-coding genes were annotated. BUSCO analysis indicated approximately 84% completeness based on core eukaryotic single-copy genes, supporting the overall completeness of the genome assembly [33]. The HMM profile of the Formin Homology 2 (FH2) domain (Pfam accession PF02181, family version 28; PF02181.28) was obtained from the Pfam database (http://pfam.xfam.org/), accessed on 6 January 2026. The hmmsearch program implemented in HMMER 3.0 was used to search for Formin-related protein sequences in *N. yezoensis*, with an E-value threshold of 1 × 10^−5^ [34]. In addition, BLAST searches against the *N. yezoensis* reference genome (taxid: 2788) were performed using the NCBI BLASTP web interface (NCBI, Bethesda, MD, USA; accessed on 2 September 2026) with Formin homologous sequences from multiple species (listed in Supplementary Table S1) as queries to complement and validate the HMM-based identification. Candidate protein sequences were subsequently validated using Pfam and the National Center for Biotechnology Information (NCBI) Conserved Domain Database (CDD) (http://www.ncbi.nlm.nih.gov/Structure/cdd/wrpsb.cgi), accessed on 20 April 2026. For CDD analysis, the CDD-62456 PSSMs database was used with an E-value cutoff of 0.01; composition-based statistics adjustment was enabled, low-complexity filtering was disabled, and the maximum number of hits was set to 500. The resulting candidate sequences were further examined for the presence of the FH2 domain, and sequences lacking the characteristic FH2 domain were excluded. Finally, the NpyFormin family members were identified.

### 2.3. Gene Structure and Protein Sequence Analysis

TBtools (2.303), an integrative toolkit for biological data analysis and visualization, was used to visualize the Formin gene family members, predict gene structures, and calculate the physicochemical properties of the Formin proteins, including molecular weight, theoretical isoelectric point (pI), instability index, aliphatic index, and grand average of hydropathicity (GRAVY) [35]. The MEME Suite (https://meme-suite.org/meme/), accessed on 13 May 2026, was used to identify conserved motifs in the Formin proteins. The analysis was performed using the “any number of repetitions” (ANR) distribution model, with the number of motifs set to nine and the motif width constrained to 16–50 amino acids. The identified motifs were subsequently visualized using TBtools. Secondary structure prediction of the Formin gene family was performed using SOPMA. Transmembrane structure prediction of gene family members was performed using TMHMM (TMHMM 2.0—DTU Health Tech—Bioinformatic Services). Subcellular localization of NpyFormins was predicted using Cell-PLoc 2.0 (http://www.csbio.sjtu.edu.cn/bioinf/Cell-PLoc-2/) and WoLF PSORT (https://www.genscript.com/wolf-psort.html), both accessed on 12 April 2026. For Cell-PLoc 2.0, the plant protein localization prediction module was used. For WoLF PSORT, the organism type was set to Plant, while the other parameters were kept at their default settings. When the two predictors yielded consistent results, the shared prediction was used as the final localization. In cases of disagreement, the prediction scores were further considered, and the higher-scoring prediction was selected as the final localization. Protein structures were modeled using the SWISS-MODEL web server, accessed on 12 May 2026, using the default modelling settings. Ligand-binding sites were predicted from the three-dimensional protein structures using P2Rank [36].

### 2.4. Prediction of Cis-Acting Elements in the Promoter Regions of Formin Genes

For cis-acting element analysis, a 2000 bp upstream region of each NpyFormin gene was extracted and analyzed using the PlantCARE database (http://bioinformatics.psb.ugent.be/webtools/plantcare/html/), accessed on 12 April 2026 [37]. The 2000 bp upstream regions were examined against the genome annotation to assess potential overlaps with neighboring genes or other annotated genomic features. A 439 bp portion of the NpyFormin02 upstream region overlapped with a neighboring annotated gene. Therefore, the predicted cis-acting elements were interpreted as elements identified within the defined 2000 bp upstream regions rather than as exclusively promoter-specific elements.

### 2.5. Phylogenetic Analysis of the Formin Gene Family

In this study, Formin family protein sequences from *N. yezoensis* (Npy), *Saccharomyces cerevisiae* (Sc), *Gracilariopsis lemaneiformis* (Gl), *Phaeodactylum tricornutum* (Pt), *A. thaliana* (At), and the marine green alga *Chloropicon primus* (Cp) were integrated to investigate the phylogenetic relationships of the Formin family. Using the same gene-family identification strategy described above, the Formin family protein sequences of *G. lemaneiformis*, *P. tricornutum*, and *C. primus* were identified from the protein sequence datasets downloaded from NCBI. The Formin protein sequence of *S. cerevisiae* was downloaded from the Saccharomyces Genome Database (SGD, https://www.yeastgenome.org/), accessed on 2 April 2026 [38]. The Formin family protein sequences of *A. thaliana* were retrieved according to the database information and methods described in reference [27]. The accession numbers of all protein sequences used for phylogenetic analysis are provided in Supplementary Table S1. Multiple sequence alignment was performed before phylogenetic reconstruction. A phylogenetic tree was constructed using the neighbor-joining (NJ) method implemented in MEGA 7.0.26 [39]. The evolutionary distances were calculated using the Poisson correction model based on amino acid substitutions. The analysis was performed assuming uniform rates among sites and homogeneous evolutionary rates among lineages. Gaps and missing data were treated using the complete deletion option. The reliability of the phylogenetic tree was evaluated using 1000 bootstrap replicates. Visualization was performed using the iTOL program (https://itol.embl.de/), accessed on 20 April 2026.

### 2.6. Environmental Treatments

In this study, the expression response patterns of Formin gene family members in *N. yezoensis* under different environmental conditions were analyzed. Temperature, light intensity, and desiccation/rehydration treatments were conducted as independent experiments. For each treatment group, three independent culture vessels were established, with each vessel defined as one biological replicate. Healthy *N. yezoensis* thalli of approximately 60 days of age with similar growth conditions were selected from the same culture batch, and an equal number of individuals were randomly assigned to the respective culture containers.

Before treatment, the thalli were maintained under a 12 h light/12 h dark photoperiod at the standard culture conditions. For different temperature treatments (4, 10, and 24 °C) [40], light intensity treatments (20, 60, and 100 μmol photons m^−2^ s^−1^) experiments, the treatments were initiated at the beginning of the light period and continued for 6 h [41,42,43]. After treatment, 0.1 g of thallus samples were taken from each culture vessel for RNA extraction (each sample was a mixture containing multiple thalli).

For the desiccation and rehydration treatments (80% desiccation and rehydration for 30 min after 80% desiccation), approximately three 0.1 g aliquots of *N. yezoensis* thalli from each group were placed in an incubator at 10 ± 1 °C under a light intensity of 60 μmol photons·m^−2^·s^−1^. The thalli were uniformly spread to facilitate even water loss. The duration of desiccation was adjusted according to the target moisture levels calculated using the formula described below. Meanwhile, approximately three 0.1 g samples of *N. yezoensis* thalli were maintained in PES medium under the same conditions (10 ± 1 °C, 60 μmol photons·m^−2^·s^−1^) as the control group, with sampling conducted at the same time points as the 80% desiccation group.

The desiccation rate of *N. yezoensis* was calculated according to the following formula:

$$WL(\%)=\frac{FW-TW}{FW-DW}\times 100$$

where *WL* is the thallus desiccation rate; *FW* is the fresh weight of the thallus; *TW* is the thallus weight after desiccation; and *DW* is the weight of fresh thallus dried at 60 °C until no further weight change was observed [44].

After completing the above treatments for each group, photographs of the algal thalli and their cells were taken respectively (see Supplementary Figures).

### 2.7. Quantitative Expression Analysis of Genes

After treatment, thalli were immediately collected, frozen in liquid nitrogen, and stored at −80 °C until RNA extraction. Total RNA was extracted using the E.Z.N.A.^®^ Plant RNA Kit (R6827-01, Omega, Norcross, GA, USA). Reverse transcription was performed using the PrimeScript^TM^ RT Reagent Kit with gDNA Eraser (No. RR047A, TaKaRa, Kusatsu, Shiga, Japan) to obtain cDNA from the relevant samples.

Quantitative real-time PCR (qRT-PCR) was performed using a Quant Gene 9600 Real-Time PCR Detection System (Bioer Technology, Hangzhou, China). Each reaction was performed in a total volume of 20 μL, containing 10.0 μL of ChamQ SYBR Color qPCR Master Mix (Q411-02/03, Vazyme, Nanjing, China), 0.4 μL of forward primer, 0.4 μL of reverse primer, 1.0 μL of cDNA template, and 8.2 μL of nuclease-free water. Each cDNA sample was analyzed using three technical replicates. The detailed qRT-PCR amplification conditions are provided in Supplementary Table S2. The amplification program consisted of an initial denaturation at 95 °C for 30 s, followed by 40 cycles of denaturation at 95 °C for 10 s and annealing/extension at 60 °C for 30 s. A melting-curve analysis from 60 to 95 °C was performed after amplification to verify the specificity of the PCR products.

Primers were designed using Primer Premier 5, and the primer sequences are listed in Table 1. The amplification efficiencies of all target and reference gene primers were determined using standard curve analysis. The amplification efficiencies were 102% for NpyFormin01, 95% for NpyFormin02, 92% for NpyFormin03, 99% for NpyUBC, and 95% for NpyMAP. The R^2^ values for NpyFormin01, NpyFormin02, and NpyFormin03 were 0.999, 0.9996, and 0.9996, respectively. The detailed amplification efficiencies and correlation coefficients (R^2^) are provided in Supplementary Table S3.

NpyUBC (ubiquitin-conjugating enzyme) and NpyMAP (methionyl aminopeptidase) were selected as reference genes according to previous studies [13]. To further evaluate their suitability under the experimental conditions of this study, the Ct values of NpyUBC and NpyMAP were examined across the temperature, light-intensity, desiccation, and rehydration treatments. Both reference genes showed relatively stable Ct distributions across the different treatments, with the corresponding mean Ct values and variation presented in Supplementary Table S4. These results supported the suitability of NpyUBC and NpyMAP as reference genes under the experimental conditions examined in this study. To reduce potential normalization bias associated with the use of a single reference gene, the RQ values of NpyUBC and NpyMAP were combined using their geometric mean to generate a normalization factor.

Relative transcript abundance was calculated using an efficiency-corrected quantitative PCR method. The mean Ct value of each sample was calculated from three technical replicates. Relative quantity (RQ) values were calculated according to the following equation:

RQ = E^ΔCt^

where E represents the amplification efficiency of each primer. The ΔCt value was calculated as the difference between the minimum Ct value observed among all samples and the Ct value of each sample. The RQ values of NpyUBC and NpyMAP were combined using their geometric mean as the normalization factor. The relative expression level of each target gene was calculated as the ratio of the target gene RQ value to the normalization factor [45].

### 2.8. Statistical Analysis

All experimental data were analyzed using SPSS 26.0. Statistical analyses were performed based on three biological replicates for each treatment. For experiments involving multiple treatment groups, one-way analysis of variance (ANOVA) was used to assess overall differences among groups, followed by Tukey’s honestly significant difference (HSD) test for post hoc pairwise comparisons while controlling for multiple comparisons. Statistical significance was defined as *p* < 0.05. In the figures, asterisks indicate statistically significant differences between groups (* *p* < 0.05) [46].

## 3. Results

### 3.1. Identification and Gene Structure Analysis of the Formin Gene Family

Through Hmmsearch retrieval and domain prediction using CDD and Pfam databases, a total of three Formin members were identified in *N. yezoensis*, namely KAK1868125.1, KAK1868180.1, and KAK1867430.1, which were designated as NpyFormin01, NpyFormin02, and NpyFormin03, respectively. All three Formin family members were located on the negative strand. The lengths of their coding sequences (CDS) showed considerable variation. Among them, NpyFormin01 had the longest CDS of 4845 bp, whereas NpyFormin02 had the shortest CDS of 2301 bp. The CDS sequence length of NpyFormin03 was 2349 bp (Table 2). The intron and exon compositions of the Formin genes in *N. yezoensis* also differed. Specifically, NpyFormin01 and NpyFormin02 exhibited a structure with zero introns and one exon. In contrast, NpyFormin03 contained one intron and two exons, which is markedly distinct from the other two members in terms of gene structure.

### 3.2. Motif and Conserved Domain Analysis of Formin Gene Family Members in N. yezoensis

**Figure 1:**
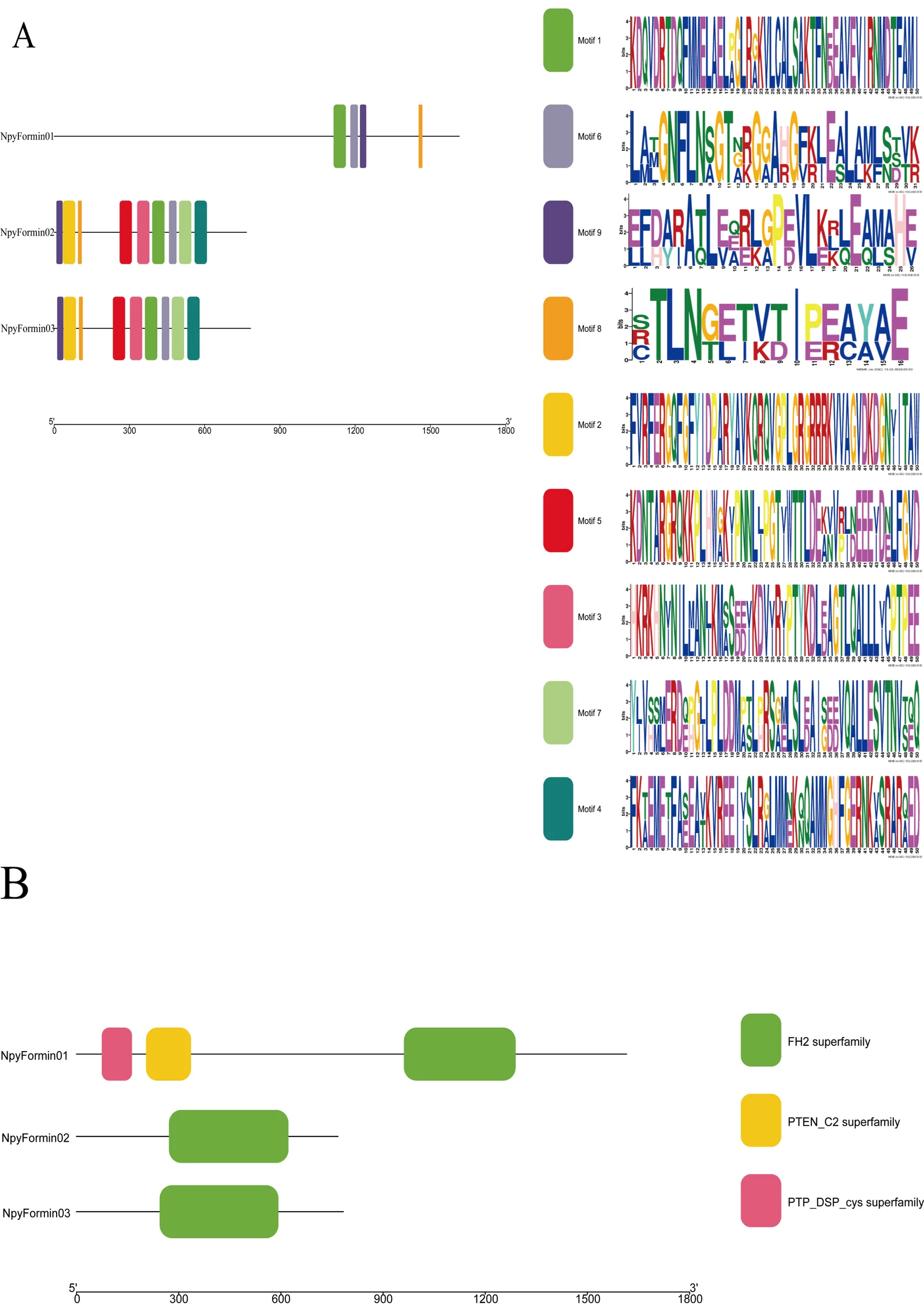
Motif composition and conserved domain analysis of Formin proteins in *N. yezoensis*. (**A**) Motif composition of Formin structures in *N. yezoensis*. (**B**) Conserved domain analysis of Formins in *N. yezoensis*.

Motif analysis revealed that all three Formin proteins of *N. yezoensis* contained Motif1, Motif6, Motif8, and Motif9, indicating that these motifs may represent conserved functional units shared among members of this family (Figure 1A). Notably, NpyFormin02 and NpyFormin03 exhibited highly consistent motif compositions and arrangements, both containing all nine predicted motifs (Motif1–Motif9), with the relative positions of each motif in the protein sequences being essentially identical. In contrast, only Motif1, Motif6, Motif8, and Motif9 were detected in NpyFormin01, and these motifs were predominantly concentrated in the C-terminal region of the protein sequence. This indicates that although NpyFormin01 retains the core conserved motifs of the Formin family, it exhibits marked differences from NpyFormin02 and NpyFormin03 in terms of both motif number and arrangement pattern.

Domain analysis showed that all three Formin proteins contained a typical FH2 superfamily domain (Figure 1B). The FH2 domain is the core domain through which Formin proteins regulate actin nucleation and elongation; therefore, this result further supports that all three sequences belong to the Formin family. In addition, NpyFormin01 contains a PTEN_C2 superfamily domain and a PTP_DSP_cys superfamily domain in its N-terminal region in addition to the FH2 domain. In contrast, only the FH2 domain was detected in NpyFormin02 and NpyFormin03, with no obvious PTEN-related domain identified. These results suggest that NpyFormin01 may possess a more complex domain architecture compared to NpyFormin02 and NpyFormin03, but further experimental validation is required.

### 3.3. Physicochemical Properties of the Formin Gene Family in N. yezoensis

The three Formin family members differed in their physicochemical properties, including the number of amino acids, molecular weight, theoretical isoelectric point (pI), and instability index, while showing consistency in hydrophilicity/hydrophobicity (Table 3). Among them, NpyFormin01 had the highest number of amino acids (1178) and consequently the largest molecular weight (123.65 kDa). NpyFormin02 and NpyFormin03 had similar amino acid numbers and their molecular weights were also close to each other, being 76.99 kDa and 79.81 kDa, respectively. The theoretical isoelectric points (pI) of all three Formins were below 7, indicating that they are predicted to be acidic proteins. Among them, NpyFormin02 had the lowest pI (5.00), NpyFormin03 had the highest pI (6.01), and NpyFormin01 had an intermediate pI (5.44).

The three Formins showed distinct differences in protein stability and hydrophobicity. The instability index of NpyFormin03 was higher than those of NpyFormin01 and NpyFormin02. NpyFormin02 had an instability index below 40, indicating relatively high protein stability. Regarding the aliphatic index, NpyFormin01 had a higher value than the other two members, while NpyFormin02 and NpyFormin03 had similar values. The GRAVY values of all three proteins were negative, indicating that all three proteins were predicted to be hydrophilic, with NpyFormin03 being the most hydrophilic.

Transmembrane domain prediction showed that all three Formin proteins had zero transmembrane helices and no hydrophobic transmembrane regions. Subcellular localization prediction indicated that the predicted localizations of the three Formin proteins differed, with NpyFormin01 predicted to be localized in the chloroplast, whereas NpyFormin02 and NpyFormin03 were predicted to be localized in the nucleus.

### 3.4. Multi-Level Structural Characteristics of Formin Gene Family Members in N. yezoensis

The predicted secondary structures of the three Formin family members in *N. yezoensis* revealed that the overall spatial conformation of all three proteins is primarily composed of α-helices as the main structural framework, supplemented by a certain proportion of random coils and a small proportion of extended strands, collectively forming the three-dimensional structural scaffold (Table 4). Among them, NpyFormin01 exhibited the highest proportion of α-helices, whereas NpyFormin02 and NpyFormin03 had lower similar α-helix proportions. NpyFormin01 had the lowest proportion of random coils, while NpyFormin02 and NpyFormin03 had higher random coil proportions. The proportions of extended strands were low for all three proteins, with only minor differences among them (Table 4).

**Figure 2:**
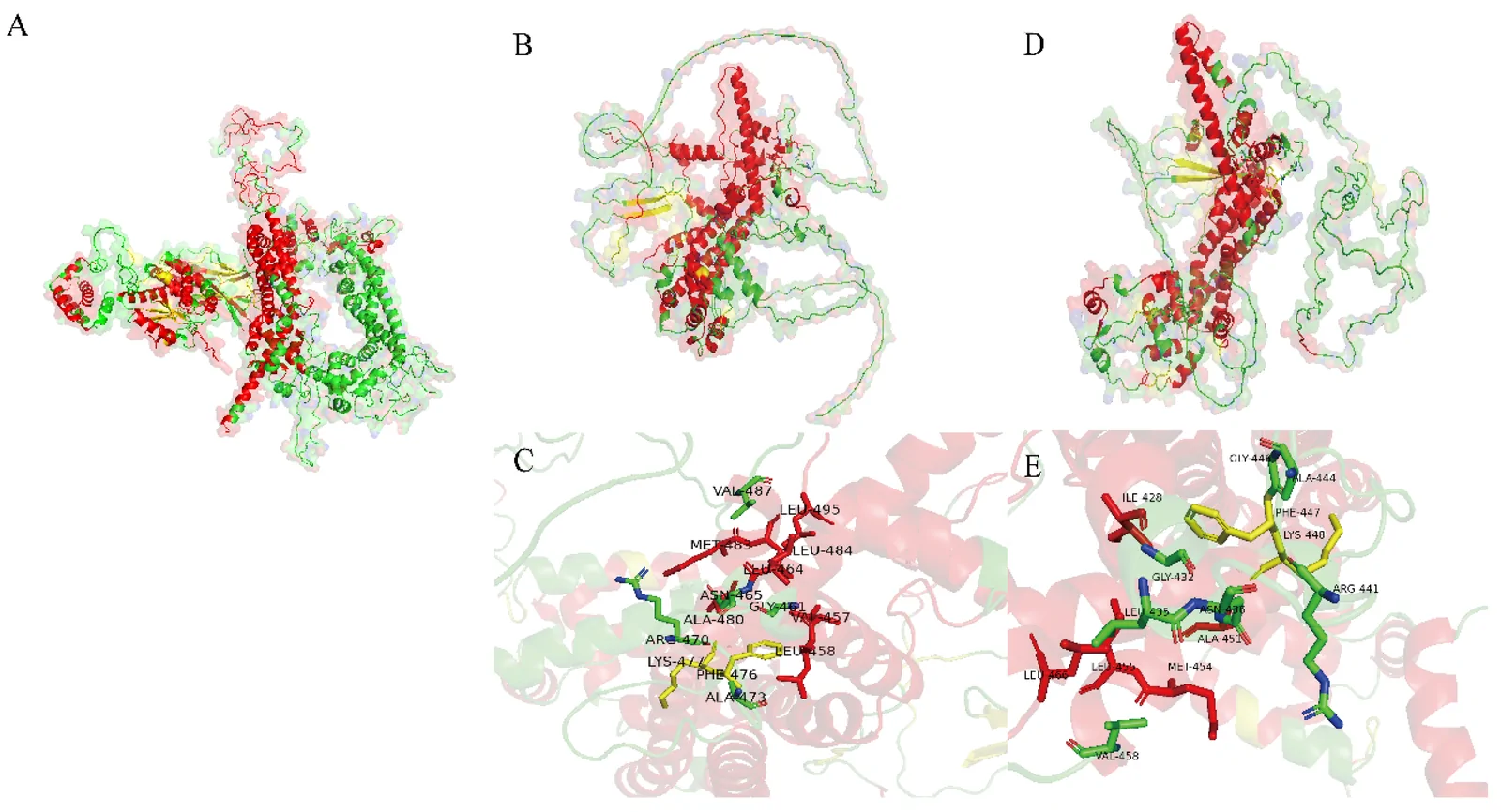
Tertiary structure prediction of the Formin gene family in *N. yezoensis*. (**A**) Predicted tertiary structure of NpyFormin01. (**B**) Predicted tertiary structure of NpyFormin02. (**C**) Predicted ligand binding sites of NpyFormin02. (**D**) Predicted tertiary structure of NpyFormin03. (**E**) Predicted ligand binding sites of NpyFormin03. Red, α-helix; yellow, extended strand; green, random coil.

Tertiary structure prediction results showed that all three NpyFormin proteins formed relatively complete spatial conformations, with obvious differences among the members. NpyFormin01 exhibited a larger overall spatial structure with multiple distinct folded regions, indicating a relatively complex spatial conformation. In contrast, NpyFormin02 and NpyFormin03 displayed more compact overall structures, and their spatial conformations were highly similar to each other (Figure 2A,B,D).

To further compare the structural features and potential interaction regions among NpyFormin members, P2Rank was used to predict ligand-binding pockets based on the predicted three-dimensional protein structures. The prediction results showed that both NpyFormin02 and NpyFormin03 contained multiple predicted ligand-binding pockets, with Pocket 1 ranking highest in both proteins and showing relatively high P2Rank scores and probabilities. For NpyFormin02, Pocket 1 had a P2Rank score of 13.18 and a probability of 0.697 and consisted of 14 residues: VAL-457, LEU-458, GLY-461, LEU-464, ASN-465, ARG-470, ALA-473, PHE-476, LYS-477, ALA-480, MET-483, LEU-484, VAL-487, and LEU-495 (Figure 2C). For NpyFormin03, Pocket 1 had a P2Rank score of 14.91 and a probability of 0.746 and also consisted of 14 residues: ILE-428, GLY-432, LEU-435, ASN-436, ARG-441, ALA-444, GLY-446, PHE-447, LYS-448, ALA-451, MET-454, LEU-455, VAL-458, and LEU-466 (Figure 2E). These high-ranked pockets were mainly located around the junctions between α-helical and coil regions and formed surface depressions with structural features characteristic of predicted ligand-binding pockets. In comparison, the remaining predicted pockets showed substantially lower probabilities, indicating relatively lower prediction confidence. The complete P2Rank prediction results, including the scores, probabilities, and corresponding residues for each predicted pocket, are provided in Supplementary Tables S5 and S6. It should be noted that these results are derived from computational predictions and thus identify predicted interaction regions, which require experimental validation to confirm actual ligand-binding sites.

### 3.5. Chromosomal Localization

**Figure 3:**
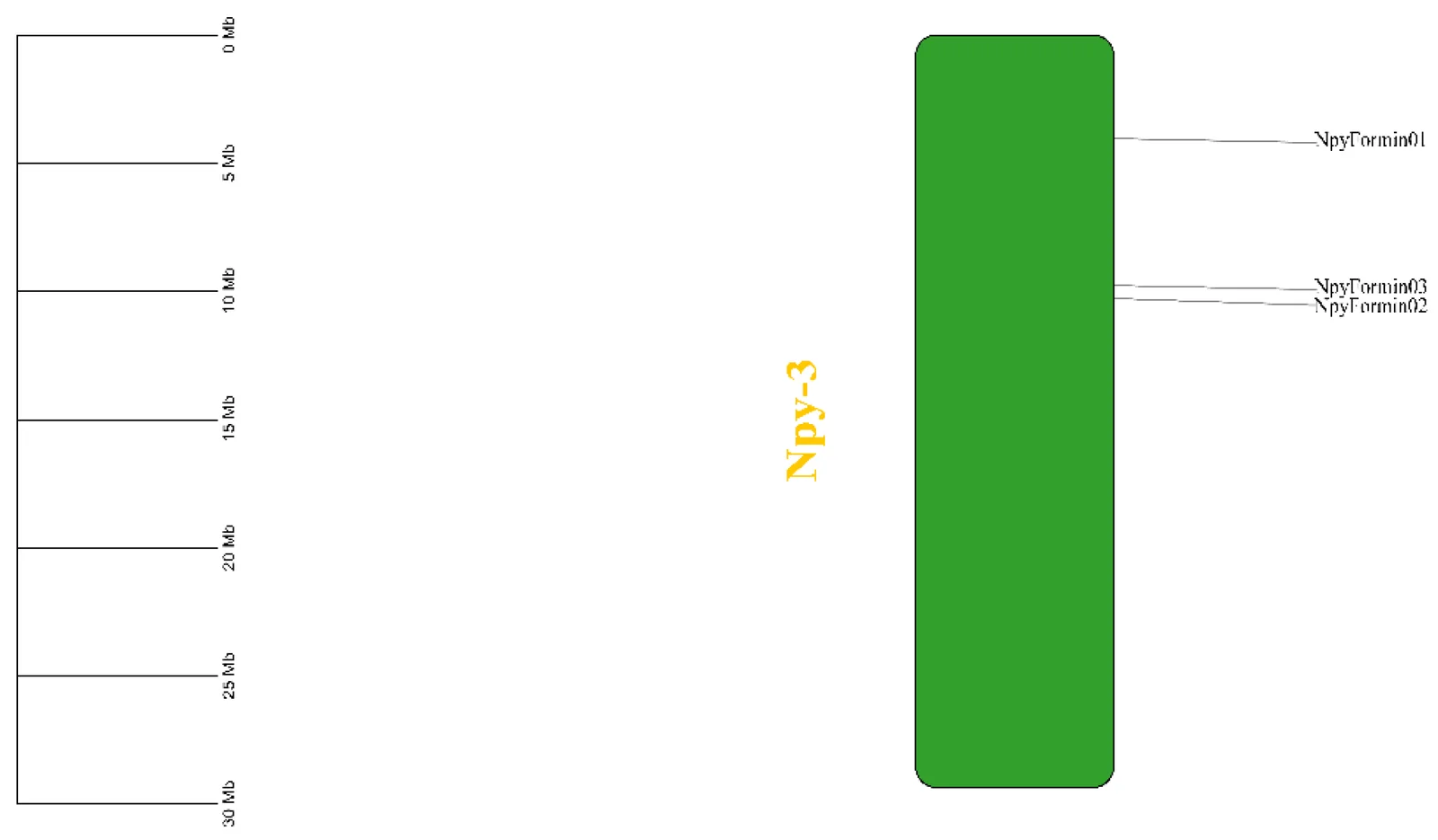
Chromosomal distribution of the Formin gene family in *N. yezoensis*.

Chromosomal localization results (Figure 3) showed that all three Formin family genes in *N. yezoensis* were localized on chromosome Npy-3, which has a total length of approximately 30 Mb. NpyFormin01 is located on chromosome Npy-3 at positions 4,038,548–4,041,273 bp. NpyFormin02 and NpyFormin03 are also located on the same chromosome (Npy-3), at positions 9,769,093–9,773,937 bp and 10,280,940–10,283,240 bp, respectively.

### 3.6. Phylogenetic Analysis

In this study, Formin family protein sequences from *N. yezoensis* (Npy), *S. cerevisiae* (Sc), *G. lemaneiformis* (Gl), *P. tricornutum* (Pt), *A. thaliana* (At), and the marine green alga *C. primus* (Cp) were integrated to construct a phylogenetic tree and systematically analyze the evolutionary relationships among Formin family members from different species.

**Figure 4:**
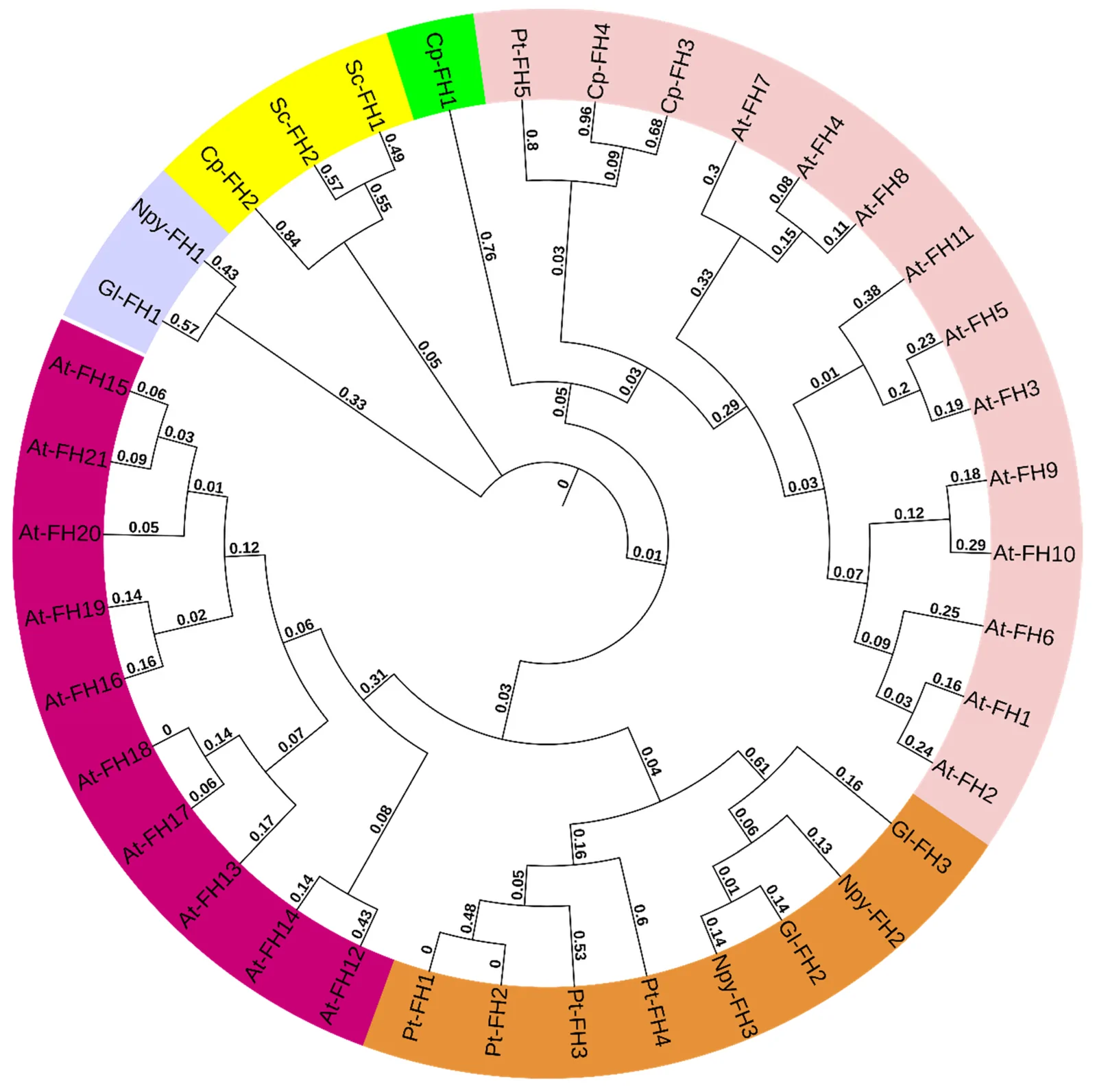
Phylogenetic analysis of the Formin gene family. Npy-FH, Sc-FH, Gl-FH, Pt-FH, At-FH, and Cp-FH represent Formins from *N. yezoensis* (Npy), *S. cerevisiae* (Sc), *G. lemaneiformis* (Gl), *P. tricornutum* (Pt), *A. thaliana* (At), and *C. primus* (Cp), respectively. The numbers in the figure represent branch lengths.

Phylogenetic analysis showed that Formin proteins from different species were clustered into five main branches (Figure 4). The three NpyFormin members of *N. yezoensis* were distributed across different branches, indicating certain differences in their sequence compositions. The number of Formin genes was small in fungi and algae, whereas in the land plant *A. thaliana*, 21 Formin members were identified, suggesting a possible expansion of the Formin family in land plants relative to algae and fungi.

Npy-FH1 clustered closely with Gl-FH1 from red algae, and together with Cp-FH2, Sc-FH1, and Sc-FH2, they formed a larger clade. This branch included Formin proteins from red algae (*N. yezoensis* and *G. lemaneiformis*), green algae *(C. primus*), and fungi (*S. cerevisiae*), and was relatively distant from the Formin proteins of land plant origin. Npy-FH2 and Npy-FH3 clustered together with Gl-FH2, Gl-FH3, and Pt-FH1–Pt-FH4 to form a single larger branch, suggesting that these Formin proteins are relatively conserved across red algae and diatoms.

### 3.7. Cis-Acting Element Analysis

**Figure 5:**
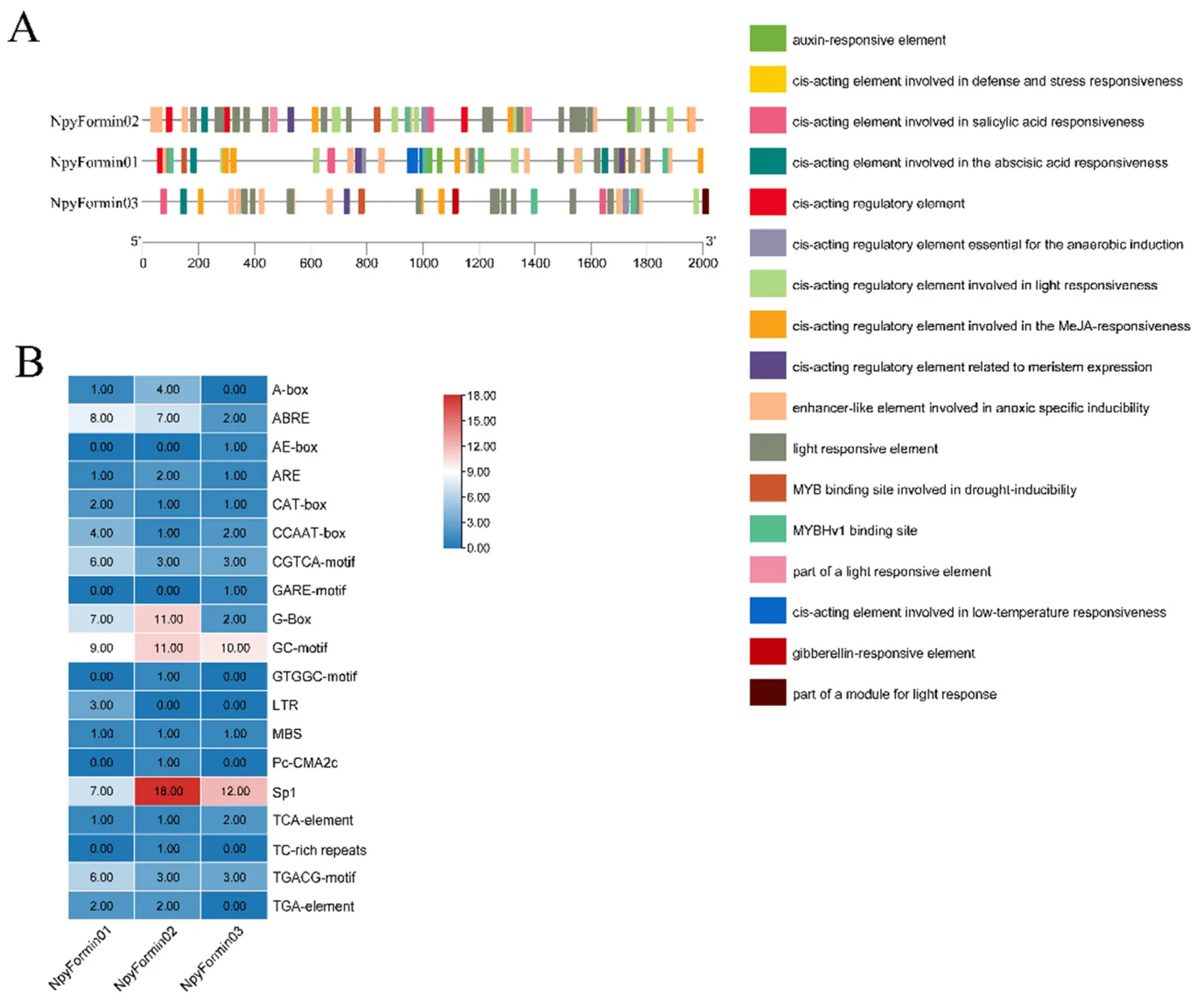
Cis-acting elements of the Formin gene family in *N. yezoensis*. (**A**) Distribution of cis-acting element functions in Formin genes of *N. yezoensis*. (**B**) Abundance of cis-acting elements in the promoter regions of Formin genes in *N. yezoensis*. The numbers indicate the counts of elements.

To gain insight into the potential regulatory mechanisms, we performed cis-acting element analysis of the 2000 bp upstream regions of the three Formin genes in *N. yezoensis*. A total of 167 predicted cis-acting elements were identified, involving various types related to light response, phytohormone response, environmental response, and basal transcriptional regulation. Among them, the 2000 bp upstream region of NpyFormin01 contained 58 predicted elements representing 14 different types, NpyFormin02 contained 68 elements representing 15 different types, and NpyFormin03 contained 41 elements representing 12 different types (Figure 5A).

Predicted light-responsive elements were the most abundant and diverse type of cis-acting elements identified within the 2000 bp upstream regions of the three Formin genes, mainly including G-box, Sp1, AE-box, GTGGC-motif, and Pc-CMA2c. Regarding distribution, the 2000 bp upstream region of NpyFormin01 contained seven G-box and seven Sp1 elements, totaling 14. NpyFormin02 had the most abundant predicted light-responsive elements, including 11 G-box, 18 Sp1, one GTGGC-motif, and one Pc-CMA2c, totaling 31. The 2000 bp upstream region of NpyFormin03 contained two G-box, 12 Sp1, and one AE-box, totaling 15 (Figure 5B).

Among the temperature-responsive elements, only LTR elements were detected, and their distribution and abundance exhibited strong gene specificity. Specifically, LTR elements were detected only in NpyFormin01 (three copies), but were absent in both NpyFormin02 and NpyFormin03 (Figure 5B).

In addition to abiotic environmental response elements associated with light, temperature, and drought, phytohormones play critical roles in regulating gene expression and stress adaptation. Our analysis revealed that the 2000 bp upstream regions of the three Formin genes contained various predicted hormone-responsive elements involved in the signaling pathways of abscisic acid (ABA), methyl jasmonate (MeJA), auxin, gibberellin, and salicylic acid (SA) (Figure 5B). Among these, ABA-responsive elements (ABRE) were the most abundant, followed by MeJA-responsive elements (CGTCA-motif and TGACG-motif), which were also widely distributed across the three upstream regions. Auxin-responsive elements (TGA-element) and SA-responsive elements (TCA-element) were present at lower frequencies. In contrast, the gibberellin-responsive element (GARE-motif) was detected exclusively in NpyFormin03. These results suggest that the three Formin genes may be associated with distinct potential phytohormone-responsive regulatory patterns.

### 3.8. Gene Expression Analysis

**Figure 6:**
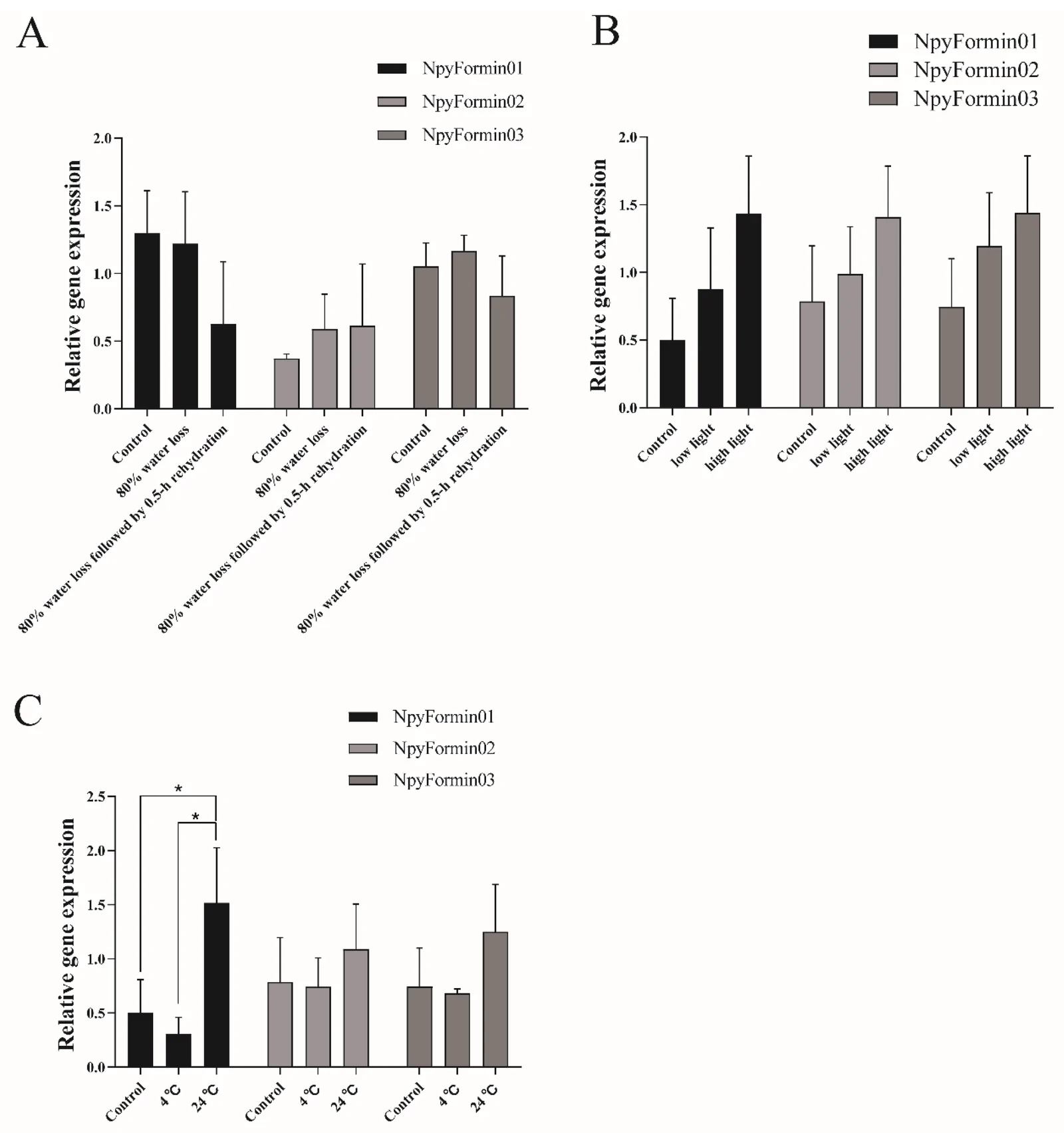
Expression analysis of Formin genes in *N. yezoensis* under different environmental conditions. (**A**) Relative expression levels of Formin genes under desiccation and subsequent rehydration. (**B**) Relative expression levels of Formin genes after 6 h exposure to different light intensities. (**C**) Relative expression levels of Formin genes after 6 h exposure to different temperatures. Statistical significance was determined using one-way analysis of variance (ANOVA) followed by Tukey’s multiple comparison test. Asterisks indicate significant differences between the indicated groups (*p* < 0.05).

To investigate the transcriptional responses of Formin genes to water deficit, the relative expression levels of NpyFormin01, NpyFormin02, and NpyFormin03 were examined under 80% desiccation and subsequent rehydration, using non-desiccated thalli as the control (Figure 6A). The three Formin genes exhibited distinct expression trends in response to desiccation and rehydration treatments, but these changes were not statistically significant.

To analyze the responses of NpyFormin genes to different light conditions, their expression levels were examined after normal light, low light, and high light treatments. All three genes showed expression variation under altered light intensities (Figure 6B). NpyFormin01, NpyFormin02, and NpyFormin03 displayed increasing trends under high light conditions compared with the control; however, the differences among treatments were not statistically significant.

To investigate the responses of NpyFormin genes to temperature changes, their expression levels were analyzed under normal-temperature, low-temperature, and high-temperature conditions. NpyFormin01 showed a significant increase in expression under high-temperature treatment compared with both the control and low-temperature groups (*p* < 0.05, Figure 6C). In contrast, although NpyFormin02 and NpyFormin03 exhibited increasing trends under high-temperature treatment, no statistically significant differences were detected among the treatments.

## 4. Discussion

In the present study, we performed a genome-wide identification and characterization of the Formin gene family in the intertidal red alga *N. yezoensis*, and examined their expression profiles under different environmental conditions. In this study, a total of three Formin members (NpyFormin01-03) were identified in *N. yezoensis*, all located on chromosome Npy-3, with marked structural divergence between NpyFormin01 and the other two isoforms. These findings, together with the differential expression patterns observed under temperature, light, and desiccation treatments, provide novel insights into the potential roles of Formins in environmental adaptation of this ecologically and economically important red alga.

The identification of only three Formin genes in *N. yezoensis* is consistent with the notion that red algal genomes possess a highly simplified cytoskeletal regulatory toolkit [47]. As noted in previous comparative genomic studies, genes encoding microfilament nucleation and polymerization factors—such as the ARP2/3 complex, WASP, SCAR, and WAVE—are markedly reduced or entirely absent in red algae, whereas Formin-encoding genes are relatively enriched [12,14]. Our phylogenetic analysis further supports this lineage-specific evolution: the three NpyFormins clustered with Formins from another red alga (*G. lemaneiformis*) and other algae/fungi, but remained distinct from the expanded Formin subfamilies in land plants such as *A. thaliana*. This phylogenetic separation, coupled with the absence of land-plant-like class I/II structural features in NpyFormin02 and NpyFormin03 [48], suggests that Formins in red algae may have undergone functional specialization distinct from their counterparts in angiosperms.

Despite the small family size, the three NpyFormins exhibited substantial differences in gene structure, protein architecture, and predicted physicochemical properties. NpyFormin01 possesses an N-terminal PTEN_C2 and PTP_DSP_cys domain in addition to the core FH2 domain, has a longer CDS (4845 bp), and is predicted to localize to the chloroplast. In contrast, NpyFormin02 and NpyFormin03 lack additional recognizable domains beyond FH2, have shorter CDSs (~2.3 kb), and are predicted to localize to the nucleus. This domain architecture dichotomy implies that NpyFormin01 may be involved in actin remodeling associated with plastid-related processes or membrane dynamics, whereas NpyFormin02 and NpyFormin03 might regulate nuclear- or chromatin-associated actin functions, as observed in some eukaryotic systems [49,50]. In the Formin gene family, high similarity in gene structure, conserved motifs, and spatial conformation, together with close chromosomal proximity, is often regarded as a potential indicator of recent gene duplication events or functional redundancy. This feature has been systematically validated in a series of studies on the Formin gene family in potato (*Solanum tuberosum* L.). Through genome-wide identification, Khatun et al. found that among the 26 Formin genes (StFH) in potato, gene structure and motif analyses both revealed high similarity within subgroups [24]. Moreover, Ka/Ks ratio analysis confirmed that two gene pairs originated from tandem duplications and five pairs from segmental duplications, with all these gene pairs undergoing purifying selection, implying functional conservation and potential redundancy [24]. Similarly, genome-wide identification of the Formin gene family in cotton (*G. hirsutum* L.) demonstrated that its 46 GhFH genes expanded through gene duplication events. Structural analysis, combined with Ka/Ks-based evolutionary selection pressure analysis, further corroborated that the duplicated gene pairs maintained highly similar motifs and structural features under purifying selection [26]. In our study, NpyFormin02 and NpyFormin03 are closely located on the same chromosome (Npy-3: positions 9,769,093–9,773,937 bp and 10,280,940–10,283,240 bp, see Results). The high similarity in their motif composition and spatial conformation, together with this close chromosomal proximity, provides indirect evidence suggestive of a potential recent duplication event, analogous to the well-characterized Formin homologs in potato and cotton [24,26]. However, we acknowledge that no formal synteny/colinearity or duplication analysis was performed in the current study. Therefore, further Ka/Ks-based selection pressure analysis and comprehensive comparative genomic analyses are required to definitively confirm their evolutionary origin and functional redundancy.

It was demonstrated by qRT-PCR that the three NpyFormin genes respond differentially to desiccation, light intensity, and temperature stress. Notably, NpyFormin01 exhibited a significant upregulation under high-temperature stress, suggesting its potential involvement in thermotolerance. Promoter sequence analysis revealed that this gene harbors multiple LTR elements. Previous studies have demonstrated that in the promoter of the HSP70 gene from *Ulva prolifera*, LTR elements not only participate in cold responses but also cooperate with heat shock elements under high-temperature stress to significantly enhance promoter transcriptional activity [51]. Based on these findings, we speculate that the LTR elements enriched in the NpyFormin01 promoter may mediate the heat-inducible expression of NpyFormin01; however, this hypothesis requires further experimental validation. In contrast, NpyFormin02 and NpyFormin03 showed moderate, non-significant increases under high temperature, implying that they may play auxiliary or condition-specific roles rather than serving as primary responders to these stresses. Under desiccation stress, none of the three genes exhibited statistically significant changes in expression. This contrasts with a recent study in *N. yezoensis*, which reported transcriptional alterations in Formin genes under similar water-deficit conditions [9]. Such discrepancy may be attributed to differences in stress intensity, exposure duration, or thallus developmental stage. Alternatively, it is possible that Formin-mediated desiccation responses operate primarily at the post-translational level—via mechanisms such as phosphorylation—rather than at the transcriptional level, as also suggested by Yin et al. (2025) [9].

It is worth noting that the Formin family identification in this study was based on the *N. yezoensis* reference genome published by Wang et al. (2020) [33], a chromosome-level assembly comprising three scaffolds totaling approximately 108 Mb with BUSCO assessment, indicating good representation of the gene space (84% complete core genes). However, approximately 48.0% of this genome consists of repetitive elements, and recent studies have highlighted that ultra-long tandem repeats and highly similar duplicated copies remain technical bottlenecks in current genome assembly, often leading to read misalignment and regional assembly gaps that compromise the accurate annotation and complete identification of genes residing in such regions [52]. Thus, while this genome represents the best available version to date, the possibility that some Formin members may have been missed or misannotated due to imperfect assembly of repetitive regions cannot be entirely excluded. Beyond genome quality, methodological constraints also merit consideration. A combined strategy of HMM searches and BLAST searches against multiple representative species (including plant, green algae, and red algae) was employed, with the presence of the conserved FH2 domain being required for final membership determination; nevertheless, this identification is acknowledged to represent a conservative set rather than an exhaustive inventory. Recent studies on large gene family evolution in Rosaceae have demonstrated that lineage-specific expansions and functional divergence can complicate cross-species identification [53], and by analogy, Formin homologs in red algae may have undergone similar divergence, rendering them less detectable using query sequences from land plants or green algae alone. The future availability of more complete genome assemblies and improved annotation strategies for *N. yezoensis* and other red algae will be essential for a more comprehensive understanding of this gene family.

## 5. Conclusions

In summary, a genome-wide identification and environmental condition-responsive expression analysis of the Formin gene family in *N. yezoensis* has been conducted. The three identified Formin genes were found to share the core FH2 domain but to differ in domain organization, structural properties, and promoter cis-element compositions, suggesting potential functional divergence. Among them, NpyFormin01 was identified as a heat-responsive candidate, whereas NpyFormin02 and NpyFormin03 were observed to exhibit stable expression across the tested stress regimes. These differential expression patterns, together with the structural differences observed among NpyFormins, are proposed to reflect distinct roles in actin cytoskeletal regulation. Through this study, the knowledge of the Formin family in red algae has not only been expanded but valuable candidate genes and experimental data have also been provided for future investigations into the molecular mechanisms underlying cytoskeletal remodeling during environmental stress responses in intertidal seaweeds.

## Figures and Tables

**Table 1:** Primer sequences used for qRT-PCR.

Gene Name	Primer Sequence (5′→3′)
NpyFormin01	F: CCAAGCAAAAGGTCCAAAAGT, R: AAACGGCTTATTGCGTTCG
NpyFormin02	F: TGGAAGCCAAGGACAATACG, R: TTGAGGTTAGCCAGCAAAATG
NpyFormin03	F: CCAAGGCGACCAAGACACTC, R: TGCGTCACGTTCGTAACCG
NpyUBC	F: CGCTGACCGTTTCCAAG, R: CGACTGCGGTTGGACTT
NpyMAP	F: TGGGTAGGAAGTGGGGCT, R: CGTGGTAGGTCGGTAGGC

**Table 2:** Gene structure characteristics of Formin family members in *N. yezoensis*.

Gene ID	Gene Name	CDS	Strand (+ −)	Intron Number	Exon Number
KAK1868125.1	NpyFormin01	4845	−	0	1
KAK1868180.1	NpyFormin02	2301	−	0	1
KAK1867430.1	NpyFormin03	2349	−	1	2

**Table 3:** Characteristics of Formin family members in *N. yezoensis*.

Gene Name	Amino Acids (aa)	Molecular Weight (kDa)	pI	Instability Index	Aliphatic Index	GRAVY	TM Helix	Subcellular Localization
NpyFormin01	1178	123.65	5.44	40.89	84.36	−0.194	0	Chloroplast
NpyFormin02	766	76.99	5	39.28	69.9	−0.317	0	Nuclear
NpyFormin03	782	79.81	6.01	48.58	68.85	−0.39	0	Nuclear

**Table 4:** Secondary structure prediction of proteins encoded by Formin gene family members in *N. yezoensis*.

Gene Name	α-Helix (%)	Random Coil (%)	Extended Strand (%)
NpyFormin01	50.85	41.09	8.06
NpyFormin02	42.69	52.61	4.7
NpyFormin03	41.94	52.43	5.63

## Data Availability

The raw genomic data of *N. yezoensis* presented in this study are openly available in [Wang et al., 2020/PRJNA591672] [33]. The original genomic data of *G. lemaneiformis*, *P. tricornutum*, and *C. primus* presented in this study are openly available in [National Center for Biotechnology Information, https://www.ncbi.nlm.nih.gov/], accessed on 20 April 2026. The original Formin protein sequence data of *S. cerevisiae* presented in this study are openly available in [Saccharomyces Genome Database, https://www.yeastgenome.org/], accessed on 2 April 2026. The original Formin protein sequence data of *A. thaliana* presented in this study are openly available in [GreenPhylDB, https://www.greenphyl.org], accessed on 2 April 2026.

## Supplementary Materials

The following supporting information can be downloaded at: https://www.mdpi.com/article/10.3390/genes17091089/s1, Figure S1: Morphological appearance of *Neopyropia yezoensis* strain LL62 under different desiccation and rehydration conditions. (A) Thalli subjected to 80% water loss; (B) thalli subjected to 80% water loss followed by 0.5 h of rehydration; and (C) control thalli without desiccation treatment. All photographs were taken under the same experimental conditions to visually document the morphological appearance of *N. yezoensis* LL62 under different water-status conditions. The ruler provides a scale reference; Figure S2: Morphological appearance of *Neopyropia yezoensis* strain LL62 under different light conditions. (A) Low-light treatment; (B) high-light treatment; and (C) control (normal-light conditions). All photographs were taken under the same experimental conditions to visually document the morphological appearance of *N. yezoensis* LL62 under different light conditions. The ruler provides a scale reference; Figure S3: Morphological appearance of *Neopyropia yezoensis* strain LL62 under different temperature conditions. (A) Low-temperature treatment; (B) high-temperature treatment; and (C) control (normal temperature conditions). All photographs were taken under the same experimental conditions to visually document the morphological appearance of *N. yezoensis* LL62 under different temperature conditions. The ruler provides a scale reference; Figure S4: Microscopic morphology of *Neopyropia yezoensis* strain LL62 under different desiccation and rehydration conditions. (A) Thalli subjected to 80% water loss; (B) thalli subjected to 80% water loss followed by 0.5 h of rehydration; and (C) control thalli without desiccation treatment. The cellular morphology of *N. yezoensis* LL62 was observed and recorded by microscopy to visually document changes in cellular structure under different desiccation and rehydration conditions. All images were acquired under the same microscopic observation conditions. Scale bars = 200 μm; Figure S5: Microscopic morphology of *Neopyropia yezoensis* strain LL62 under different light conditions. (A) Low-light treatment; (B) high-light treatment; and (C) control (normal light conditions). The cellular morphology of *N. yezoensis* LL62 was observed and recorded by microscopy to visually document the cellular structural characteristics of the thalli under different light conditions. All images were acquired under the same microscopic observation conditions. Scale bars = 200 μm; Figure S6: Microscopic morphology of *Neopyropia yezoensis* strain LL62 under different temperature conditions. (A) Low-temperature treatment; (B) high-temperature treatment; and (C) control (normal temperature conditions). The cellular morphology of *N. yezoensis* LL62 was observed and recorded by microscopy to visually document the cellular structural characteristics of the thalli under different temperature conditions. All images were acquired under the same microscopic observation conditions. Scale bars = 200 μm; Table S1: Accession numbers of Formin protein sequences used for phylogenetic analysis; Table S2: qRT-PCR amplification program; Table S3: Amplification efficiencies and correlation coefficients (R^2^) of qRT-PCR primers determined by standard curve analysis; Table S4: Ct values of NpyUBC and NpyMAP under different experimental treatments; Table S5: P2Rank-predicted ligand-binding pockets and corresponding residues of NpyFormin02; Table S6: P2Rank-predicted ligand-binding pockets and corresponding residues of NpyFormin03.
