# Supplementary material for: Identification and Expression Analysis of the Formin Gene Family in Neopyropia yezoensis Under Different Environmental Conditions
Source: Genes (Basel). 2026 Sep 10;17(9):1089. doi: 10.3390/genes17091089 (PMC13606620; doi:10.3390/genes17091089)
Supplement: Supplementary file 1 [file genes-17-01089-s001.zip › Supplementary Figures.pdf]

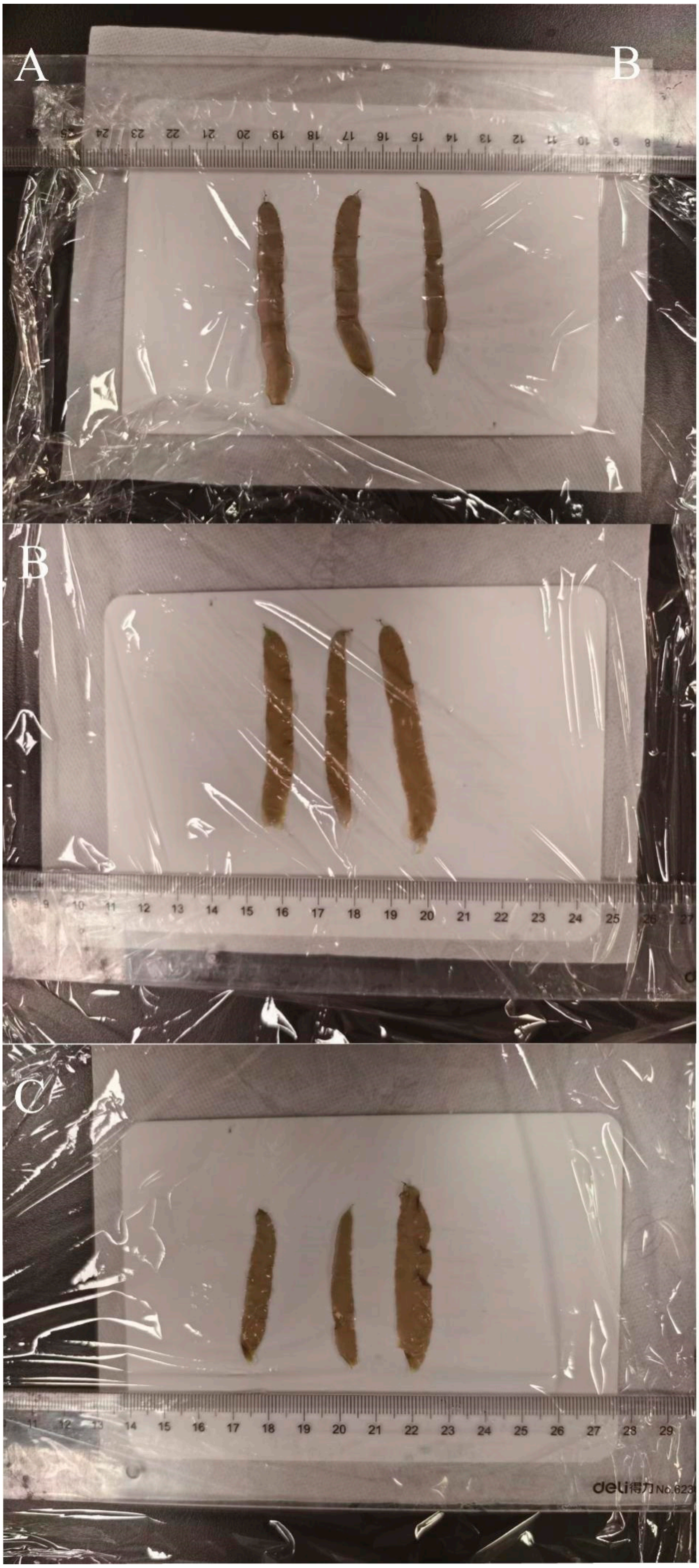
A
B
B
C
deli 得力

Figure S1. Morphological appearance of *Neopyropia yezoensis* strain LL62 under different desiccation and rehydration conditions. (A) Thalli subjected to 80% water loss; (B) thalli subjected to 80% water loss followed by 0.5 h of rehydration; and (C) control thalli without desiccation treatment. All photographs were taken under the same experimental conditions to visually document the morphological appearance of *N. yezoensis* LL62 under different water-status conditions. The ruler provides a scale reference.

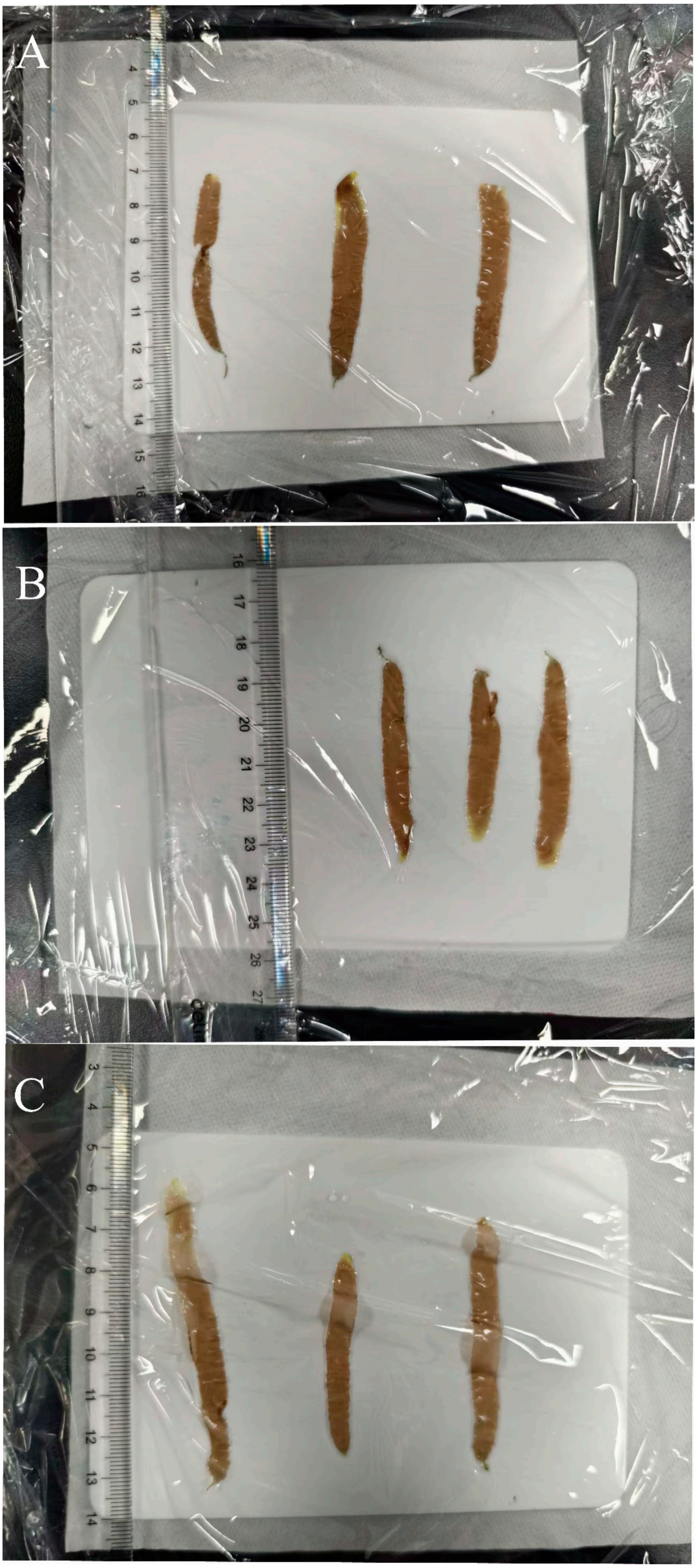
A
B
C

Figure S2. Morphological appearance of *Neopyropia yezoensis* strain LL62 under different light conditions. (A) Low-light treatment; (B) high-light treatment; and (C) control (normal-light conditions). All photographs were taken under the same experimental conditions to visually document the morphological appearance of *N. yezoensis* LL62 under different light conditions. The ruler provides a scale reference.

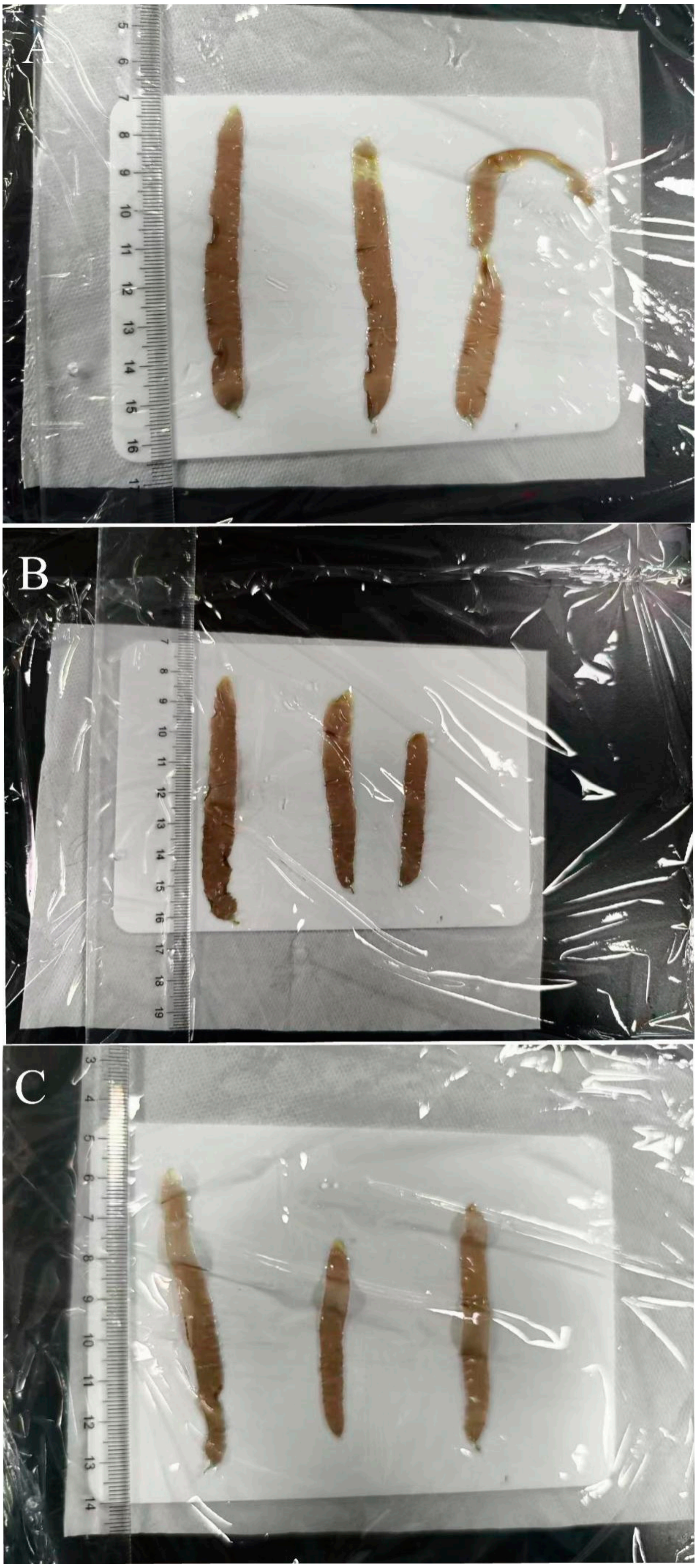
A
B
C

Figure S3. Morphological appearance of *Neopyropia yezoensis* strain LL62 under different temperature conditions. (A) Low-temperature treatment; (B) high-temperature treatment; and (C) control (normal temperature conditions). All photographs were taken under the same experimental conditions to visually document the morphological appearance of *N. yezoensis* LL62 under different temperature conditions. The ruler provides a scale reference.

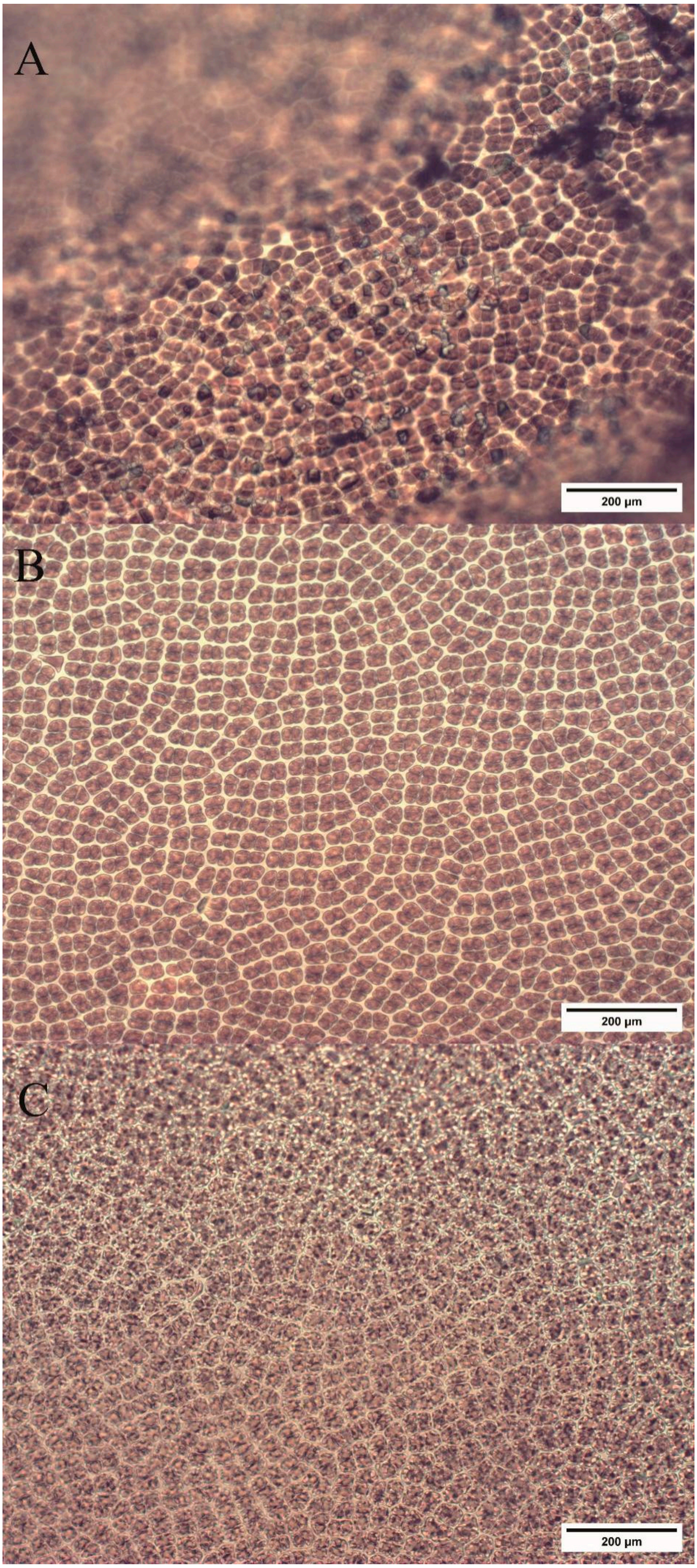
A
200 µm
B
200 µm
C
200 µm

Figure S4. Microscopic morphology of *Neopyropia yezoensis* strain LL62 under different desiccation and rehydration conditions.

(A) Thalli subjected to 80% water loss; (B) thalli subjected to 80% water loss followed by 0.5 h of rehydration; and (C) control thalli without desiccation treatment. The cellular morphology of *N. yezoensis* LL62 was observed and recorded by microscopy to visually document changes in cellular structure under different desiccation and rehydration conditions. All images were acquired under the same microscopic observation conditions. Scale bars = 200 μm.

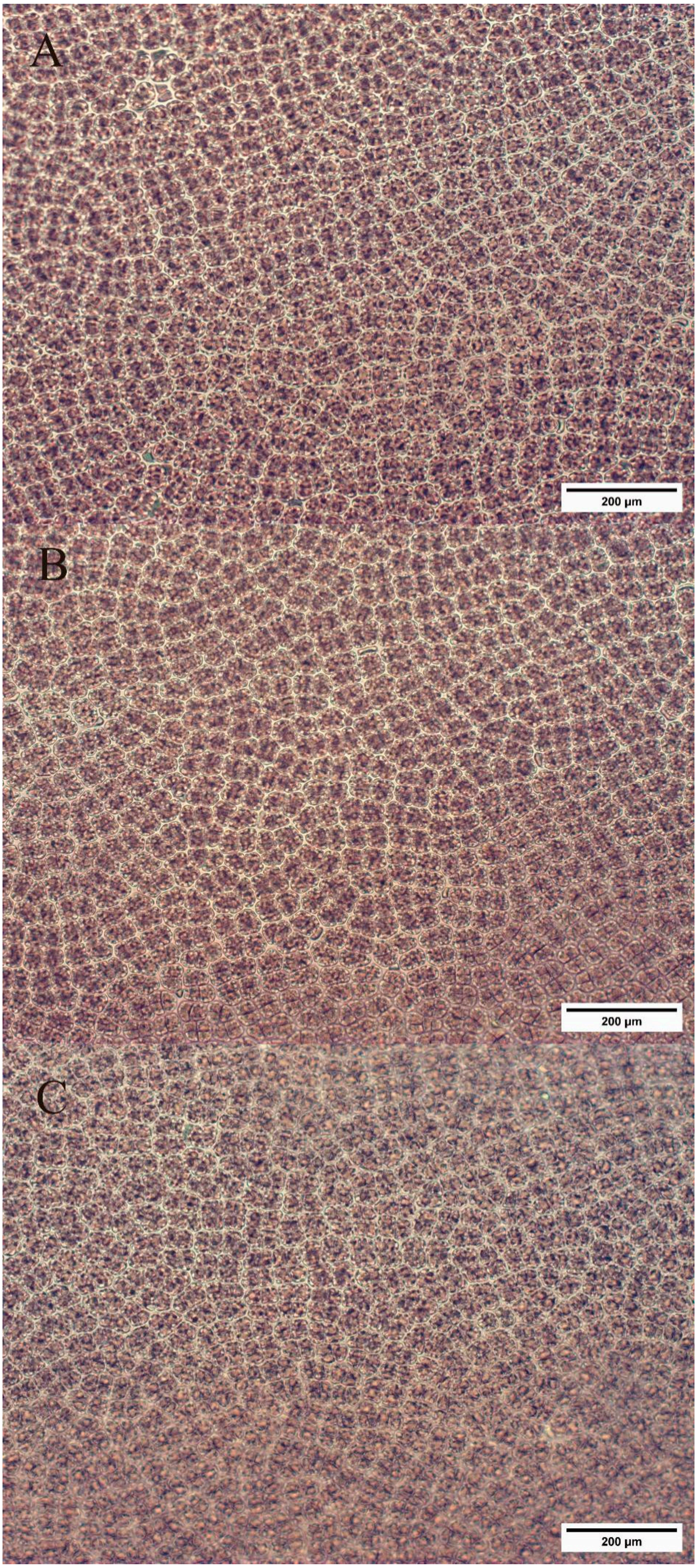
A
200 µm
B
200 µm
C
200 µm

Figure S5. Microscopic morphology of *Neopyropia yezoensis* strain LL62 under different light conditions (A) Low-light treatment; (B) high-light treatment; and (C) control (normal light conditions). The cellular morphology of *N. yezoensis* LL62 was observed and recorded by microscopy to visually document the cellular structural characteristics of the thalli under different light conditions. All images were acquired under the same microscopic observation conditions. Scale bars = 200 μm.

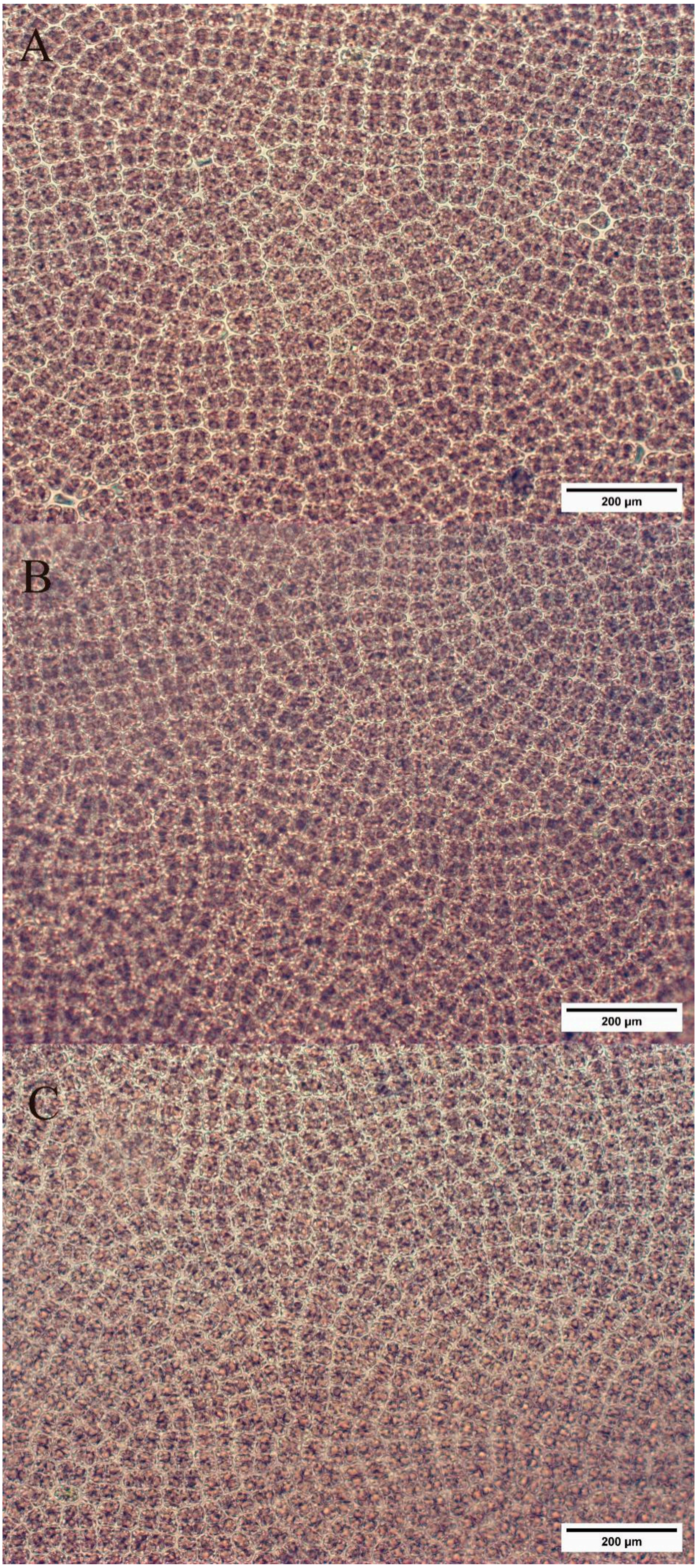
A
200 µm
B
200 µm
C
200 µm

Figure S6. Microscopic morphology of *Neopyropia yezoensis* strain LL62 under different temperature conditions. (A) Low-temperature treatment; (B) high-temperature treatment; and (C) control (normal temperature conditions). The cellular morphology of *N. yezoensis* LL62 was observed and recorded by microscopy to visually document the cellular structural characteristics of the thalli under different temperature conditions. All images were acquired under the same microscopic observation conditions. Scale bars = 200 μm.
