# Supplementary material for: Identification and Expression Analysis of the Formin Gene Family in Neopyropia yezoensis Under Different Environmental Conditions
Source: Genes (Basel). 2026 Sep 10;17(9):1089. doi: 10.3390/genes17091089 (PMC13606620; doi:10.3390/genes17091089)
Supplement: Supplementary file 1 [file genes-17-01089-s001.zip › Supplementary Table S1.pdf]

Supplementary Table S1. Accession numbers of Formin protein sequences used for phylogenetic analysis

No.	Sequence ID	Species	Sequence source/database	Amino acid sequence length (aa)	Protein sequence
1	Npy-FH1	*Neopyropia yezoensis*	This study	1178	MKGLGLLQTFYKKPVKRCVVELSDGLWAGVHSDDGAFDGDLRAVLAKS<br>RKRYGDL<br>SEILLVDTSPAPFLTSVCADVLTSHVVYLGPWAGAPSLHSVWEVVADMYD<br>YLAAG<br>AATGHSRVVLLVGTNLFPGYPAMAPVALLSAAYWVYTRVAETGTDALAL<br>FLARAR<br>SARLTTDRQLLEEADGSRRPNLTALMGWFSAVRRTASLPLRKPVRLLKVL<br>VQGSV<br>TLAGGDPWNPIVRLYTPSKFEDAHECVTLVRREGEAAAVGTDFASFDLDE<br>VVCGD<br>AVLVFEHWLPLVDQTRPLFSAVLHTSFVEAPCHRVGIGELDLAPDVGELRV<br>DSHL<br>AVDVFFPDDVDTEPEVDDTEGNAAFEKKYGAGVLHYLDRVREEASVISNL<br>ASTAP<br>TAPLVHPAHVAGAGAGGVPGSGAGAGVGMGGGPGMGGASGAAPPRYNP<br>TVTAQQA<br>AIMDSSAGQDFLSELTATVGARNATAAADAISAAGHLGDGGADDVEARR<br>KMKLIQ<br>DMLKQVPEHDLAHFAQAFHALAAAKAAKAKRNETKMVHWTPLSKQKVQ<br>KSLFESE<br>AFRNNAAADMAKLVDEGVQKQIQAAFSSKPPPKAEPTPEEAAAAAEAERN<br>KPFSA<br>GVLEARRMQNIEITLRRFPVGPPAIVDAIRCLDPTGVVLSDENVAALGATIP<br>TSE<br>ECMAAKLFAASPEGIENLTKLNPAESFVVHASKVPRFSQKVSAMLTIRSAT<br>GAAA<br>EIKKSLDTLLAACREVKASDRLQSFLAVALMTGNFLNAGTNKGAARGVRI<br>ESLLK<br>FNDTRSLDRDVSLLHYIAQLVAEKAPEVLELQEQLSHVAAAQRISKDDLSV<br>ELTQ<br>LQRAITILGSEITQMLKDEPAEAALRAAGPPPPSGASKSSTAGGLRVAPPPP<br>QQL<br>LSVVGRDLSRGTSSAGKVVGAERRAGPLASGDSLSSTSSPPTGVAEADAAA<br>AGAK<br>KPTDAAVAQGGDDTGEGDGDDVDEAGADAEETPLAAARRVYAEAESTSA<br>ELQVLL<br>NTAVREFEDTATFLGEDTRSKTDELFCTLNTLIKDIERCAVENAAREKDKA<br>KKEA<br>QKQRLAAANQAKEGKDKAITVKDAVKDAAAVKDSPATPEQVALNADRSS<br>PSSLLS<br>NTDGADAGKPTGTLPVSRVGGNGDGDVAPPEEDHVELTFGAHDGPLSSST<br>PSDSS<br>ATSSRRGSARRKRSNRTRRPAQT

No.	Sequence ID	Species	Sequence source/database	Amino acid sequence length (aa)	Protein sequence
2	Npy-FH2	*Neopyropia yezoensis*	This study	766	MARRAKAREFDARATLEERLGPDVLKRLEAMAHEFVRFERGQFGFYIDPA<br>RYAVK<br>QRQVGPLGRGRRRKVVAGVDKDGNVITAWIGVDGKAKDASTLNGETVTI<br>PEAYAE<br>GAFGIGVGPDGEGGGGGGGGGGGGGGGGGNGGGGGGGGGGGGGGGGG<br>GGGGGGGG<br>GGGDGDGGGGGPGGGGGGGPGGGGGGPGGKGPGAKAGDAGKLTGAAA<br>AVAAARQN<br>EAKRLASGKPGDPGFGGPGIGGATPGQPAAPRLPTGKVEAKDNTARGRQK<br>KPLHW<br>GKIPNNLIPGTIWTTLDEKVVPINEEEVDELFGVDVQESTGFGAAQEEVKPE<br>VLP<br>HKRKHNVNILLANLKMSSDDIKDVIRIPTYKDLEAGTLQALLLICPTPEEEQ<br>LLA<br>QNVGIKDQVDRTDQFMMELAELAGLRGKVLCALSAKTFNDEAVEVIRNM<br>DTFAMI<br>PAEVMASTKLASTLQTVLAMGNFLNSGTARGGAHGFKLEALAMLSTVKN<br>AQGGSL<br>LDYIVSSMERDQPGILPLDDMASLHRSAMLSLDAIGEDVQALLESVTNVSE<br>QIKS<br>IGDDASLASFKTEMETFASEAVKVREEIISLRGLMMEKQQAMMGHFGERN<br>KASRA<br>RAEDLFRMLREFADDVDHALMVKWEKDMRDAKKAGRAPPPKPGTRAEA<br>AAAAEAE<br>AAAAAAEMGDSPSSAAPETPSAAPVASPTPTAAAAASPTAAASTAPPAAAE<br>PTAT<br>APPADEAAAPTGAPAPDASAADAAPADTPAADVAPAADGEAAPTGDETAP<br>Q

No.	Sequence ID	Species	Sequence source/database	Amino acid sequence length (aa)	Protein sequence
3	Npy-FH3	*Neopyropia yezoensis*	This study	782	MSRRGAKGANKEFDARATLEQRLGPEVLKKLEAMAHEFVRFERGQFGFYIDPARY<br>AVKQRQVGPLGRGRRRKVVAGVDKDGNIITAWVGVDAKGGKGARTLNGETVTIPE<br>AYAEGALGGGGFGGPGGYGPGGGGGPGFGPGGGGGPGFGPGEGGGGGWGSGGPGG<br>GPGGGGPGGKGPGAAGAAAAAAAARKNQQALSGAPGAPGAPGFGGPGMAMDENGQ<br>PMAPKLPTGSVDAKDNTARGRQKKPLHWAKVPNNLLPGTVWTTLDEANVRLDEEE<br>IDNLFGVDVVERFQLQQEEVKPEVLPHKRKHNINILMANIKMASEEVKDVVRVPT<br>YKDLDAGTLQALLLVCPTPEEEELLNKNLEMKDQVDRTDQFMMELAELPGLRAKV<br>LCALSAKTFNEEAVEVIRNMDTFAMIPVEVMGSAKATKTLEAILALGNFLNSGTG<br>RGGAHGFKLEALAMLSSVKDARGGTLLDYLVHMLERDEHGLLPLDDMPTLPRSGE<br>LSLEAISDEVQALLESVTNVTQQISVIGSDGTLAVFKAEMEIFAEEATKVREEIV<br>SLRALMMNKNQAMMGHFGERNKVSRARQEDVLRMLREFLVDLDAASARLNEKRER<br>AMKTAAKAAQASGADPHEAAQSAAAAVGGYGSRHSGVTTPRAPSRLHLLNVPAAD<br>DGGDGDGGDAGGGGGGVPPPPPPMGGGGGGGGAPPPPPGMGNGDAGLSGGASAAV<br>RSMMPAAPAGGGAPPPPPPPPPRPPSVSGGGAPPPPPPPPPPPPKAAGGGAPPPP<br>PPPPPPPPPPRG

No.	Sequence ID	Species	Sequence source/database	Amino acid sequence length (aa)	Protein sequence
4	At-FH14	*Arabidopsis thaliana*	Ref. [27]	1230	MSLLSRFFYKRPPDGLLEFADRVYVFDSCFCTEVLADSLYQIFLHEVINDLH<br>EEF<br>PESSFLAFNFREGEKKSVFAETLCEYDVTVLEYPRQYEGCPMLPLSLIQHFL<br>RVC<br>ESWLARGNRQDVILLHCERGGWPLLAFILASFLIFRKVHSGERRTLEIVHRE<br>APK<br>GLLQLLSPLNPFPSQLRYLQYVARRNINSEWPPPERALSLDCVIIRGIPNFDS<br>QH<br>GCRPIIRIFGRNYSSKSGLSTEMVYSMSDKKKPLRHYRQAECDVIKIDIQCW<br>VQG<br>DVVLECVHMDLDPEREVMMFRVMFNTAFIRSNILMLNSDNLDILWEAKD<br>HYPKGF<br>RAEVLFGEVENASPQKVPTPIVNGDETGGLPIEAFSRVQELFSGVDLAENG<br>DDAA<br>LWLLKQLAAINDAKEFTRFRHKGSFYFNSPDSEEETNTSSAADSSDEGFEAI<br>QRP<br>RIHIPFDNDDTDDITLSVAHESSEEPHEFSHHHHHEIPAKDSVDNPLNLPSDP<br>PS<br>SGDHVTLLPPPPPPPPPPLFTSTTSFSPSQPPPPPPPPPLFMSTTSFSPSQPPPP<br>PPPPPLFTSTTSFSPSQPPPPPPPLPSFSNRDPLTTLHQPINKTPPPPPPPPPLP<br>SRSIPPPLAQPPPPRPPPPPPPPPSSRSIPSPSAPPPPPPPPPSFGSTGNKRQAQ<br>PPPPPPPPPPTRIPAAKCAPPPPPPPPTSHSGSIRVGPPSTPPPPPPPPPKANIS<br>NAPKPPAPPPLPPSSTRLGAPPPPPPPPLSKTPAPPPPPLSKTPVPPPPPGLGRG<br>TSSGPPPLGAKGSNAPPPPPPAGRGRASLGLGRGRGVSVPTAAPKKTALKP<br>LHWS<br>KVTRAAKGSLWADTQKQENQPRAPEIDISELESLFSAVSDTTAKKSTGRRG<br>SSIS<br>KPEKVQLVDLRRANNCEIMLTKIKIPLPDMLSAVLALDSLALDIDQVENLIK<br>FCP<br>TKEEMELLRNYTGDKEMLGKCEQFFMELMKVPRIEAKLRVFGFKITFASQ<br>VEELK<br>SCLNTINAATKEVKESAKLRQIMQTILTLGNALNQGTARGSAVGFKLDSLL<br>KLSD<br>TRARNNKMTLMHYLCKLVGEKMPELLDFANDLVHLEAASKIELKTLAEE<br>MQAATK<br>GLEKVEQELMASENDGAISLGFRKVLKEFLDMADEEVKTLASLYSEVGRN<br>ADSLS<br>HYFGEDPARCPFEQVTKILTLFMKTFIKSREENEKQAEAEKKKLEKEAIKEK<br>SAT<br>KKDGVDNDNDLIQQIHRHRT

No.	Sequence ID	Species	Sequence source/database	Amino acid sequence length (aa)	Protein sequence
5	At-FH7	*Arabidopsis thaliana*	Ref. [27]	929	MSFLFRKNGSSSRRKIKEKLRGRHSDRGGKREEDERYGGSGSDLPPPPSPWGFLF<br>PEDFVRIDGNLKAVIVDDEGLDVIYWKKLLELENSGKIRKNPKPRRRGDKSGDGF<br>RRTGADQDDNDDGDDEVGDESIEEAFSFHVKKSQSASSSGGEIRDQSNNGGGGGG<br>GGGGGGRYYTSSSASPSRPSSSSASAASPSRTSYATSAGSDYGGGGGGKQSQSKF<br>QAPGGGSFPSSPSQIHSGGGRSPPLPLPPGQFTAGNASFPSSTQPPPGQYMAGNA<br>SFPSSTPPPPGQYMAGNAPFSSSTPLPPGQYPAVNAQLSTSAPSVPLPPGQYTAV<br>NAPFSTSTQPVSLPPGQYMPGNAALSASTPLTPGQFTTANAPPAPPGPANQTSPP<br>PPPPPSAAAPPPPPPPKKGPAAPPPPPPPGKKGAGPPPPPPMSKKGPPKPPGNPK<br>GPTKSGETSLAVGKTEDPTQPKLKPLHWDKMNPDASRSMVWHKIDGGSFNFDGDL<br>MEALFGYVARKPSESNSVPQNQTVSNSVPHNQTYILDPRKSQNKAIVLKSLGMTK<br>EEIIDLLTEGHDAESDTLEKLAGIAPTPEEQTEIIDFDGEPMTLAYADSLLFHIL<br>KAVPSAFNRFNVMLFKINYGSEVAQQKGSLLTLESACNELRARGLFMKLLEAILK<br>AGNRMNAGTARGNAQAFNLTALRKLSDVKSVDAKTTLLHFVVEEVVRSEGKRAAM<br>NKNMMSSDNGSGENADMSREEQEIEFIKMGLPIIGGLSSEFTNVKKAAGIDYDSF<br>VATTLALGTRVKETKRLLDQSKGKEDGCLTKLRSFFESAEEELKVITEEQLRIME<br>LVKKTTNYYQAGALKERNLFQLFVIIRDFLGMVDNACSEIARNQRKQQQQRPATT<br>VAGASSSPAETPSVAAAPQRNAVRFPILPPNFMSESSRYSSSSDSDSES

No.	Sequence ID	Species	Sequence source/database	Amino acid sequence length (aa)	Protein sequence
6	At-FH8	*Arabidopsis thaliana*	Ref. [27]	760	MAAMFNHPWPNLTLIYFFFIVVLPFQSLSQFDSPQNIETFFPISSLSPVPPPLLP<br>PSSNPSPPSNNSSSSDKKTITKAVLITAASTLLVAGVFFFCLQRCIIARRRRDRV<br>GPVRVENTLPPYPPPPMTSAAVTTTTLAREGFTRFGGVKGLILDENGLDVLYWRK<br>LQSQRERSGSFRKQIVTGEEEDEKEVIYYKNKKKTEPVTEIPLLRGRSSTSHSVI<br>HNEDHQPPPQVKQSEPTPPPPPPSIAVKQSAPTPSPPPPIKKGSSPSPPPPPPVK<br>KVGALSSSASKPPPAPVRGASGGETSKQVKLKPLHWDKVNPDSDHSMVWDKIDRG<br>SFSFDGDLMEALFGYVAVGKKSPEQGDEKNPKSTQIFILDPRKSQNTAIVLKSLG<br>MTREELVESLIEGNDFVPDTLERLARIAPTKEEQSAILEFDGDTAKLADAETFLF<br>HLLKSVPTAFTRLNAFLFRANYYPEMAHHSKCLQTLDLACKELRSRGLFVKLLEA<br>ILKAGNRMNAGTARGNAQAFNLTALLKLSDVKSVDGKTSLLNFVVEEVVRSEGKR<br>CVMNRRSHSLTRSGSSNYNGGNSSLQVMSKEEQEKEYLKLGLPVVGGLSSEFSNV<br>KKAACVDYETVVATCSALAVRAKDAKTVIGECEDGEGGRFVKTMMTFLDSVEEEV<br>KIAKGEERKVMELVKRTTDYYQAGAVTKGKNPLHLFVIVRDFLAMVDKVCLDIMR<br>NMQRRKVGSPISPSSQRNAVKFPVLPPNFMSDRAWSDSGGSDSDM

No.	Sequence ID	Species	Sequence source/database	Amino acid sequence length (aa)	Protein sequence
7	At-FH4	*Arabidopsis thaliana*	Ref. [27]	725	MAAMLMQPWPPFLPHLTLVFLTLILFFPNQSFSQSDSPRNIETFFPNDTITPP<br>VQ<br>SPVLSPPQNPSSSSSDSDRGNILRAVLITAASTLLVAAVFFFLVHKCRRRRNR<br>VG<br>GVDNTLQPPVPPLAEAALAREGFTRFGGNVKGLILDENGLDVLYWRKLQQ<br>SQRDN<br>KGGSFRKEIIHGDDEEKNVIYSKSKKKSGPVTETPLLRGRSSTSHSVIHNDN<br>YRN<br>ATTTHPPHVKTDSFEFVKPDPTPPPPPPPPIPVKQSATPPPPPPPKLKNNGPSP<br>P<br>PPPPLKKTAALSSSASKKPPPAPRGSSSGESSNGQVKLKPLHWDKVNPDSD<br>HSMV<br>WDKIDRGSFSFDGDLMEALFGYVAVGKKSPDDGGDKKPSSASPAQIFILDP<br>RKSQ<br>NTAIVLKSLGMTRDELVESLMEGHDFHPDTLERLSRIAPTKEEQSAILQFDG<br>DTK<br>MLADAESFLFHLLKAVPCAFTRLNALLFRANYYPEISNHNKNLQTLDLACT<br>ELRS<br>RGLFSVDGKTTLLNFVVEEVVRSEGKRCVLNRRTNRSFSRSSSSSISEVISKE<br>EQ<br>EKEYLRLGLPVVGGLSSEFTNVKKAAAVDYDTVAATCLALTSRAKDARR<br>VLAQSE<br>GDNKEGVRFVKKMNEFLDSVEEEVKLAKEEEKKVLELVKRTTEYYQAGA<br>VKGKNP<br>LHLFVIVRDFLAMVDKVCVEIARNLQRRSSMGSTQQRNAVKFPVLPPNFM<br>SDRSR<br>SDSGGSDSDM
8	At-FH12	*Arabidopsis thaliana*	Ref. [27]	299	MASNCEKMLSKIKIPLPDMLNAVLDLDSSAVIIDQIKNLIKICWSKEEMDRL<br>RNS<br>AGGDKEVLGKCEEIFGELMMVPRIEPKLRVFAFKVEYPSRVSDLKMWMHT<br>IIAAT<br>KEITGSVKLFRIMQTSLTMQVLRGSNVECGLDSLVKLCDNVYLMHDFCKL<br>LDFGN<br>DLVHLEAASRIELETITNKMQELFDIEEEVNDEFLASENDGANFVGYRNVV<br>HDFL<br>CTIDGDKQLLNILYAEVGGLVNSYIAEYPSGVRFKEATNILTRFVETFYKSR<br>EEI<br>ERQAEAEKEILEKRKMNIKQNGNL

No.	Sequence ID	Species	Sequence source/database	Amino acid sequence length (aa)	Protein sequence
9	At-FH2	*Arabidopsis thaliana*	Ref. [27]	894	MTTIPFCFLFVAFFFSSSTADQRHHSRHLLHQPFFPVVTAAPPPYQPPVSSQPPS<br>PSPHTHHHHKKHLTTTTPPPHEKHLFSSVANPPPPPPSPPHPNPFFPSSDPTSTA<br>SHPPPAPPPPASLPTFPANISSLLFPTHNKQSKPPSNGHIARLVTITASVISAAA<br>LLSLFAVFIIFIRRTRHRRRSSPADDTKSTRSDALQLFNASPSDGSKKQKQHQQP<br>PKYTSSHTSSEFLYLGTLVNSRSNGLEQQKSPISLSGGITGVLELPPPASSSSSS<br>SYSQYHKLGSPELRPLPPLPKLQSFTPVYKSTEQLNPKRQDFDGDDNENDEFFSP<br>RGSSGRKQSPTRVSDVDQIDNRSINGSGSNSCSPTNFAPSLNASPGTSLKPKSIS<br>PPVSLHSQISSNNGIPKRLCPARPPPPPPPPPQVSEVPATMSHSLPGDDSDPEKK<br>VETMKPKLKTLHWDKVRASSSRVMVWDQIKSNSFQVNEEMIETLFKVNDPTSRTR<br>DGVVQSVSQENRFLDPRKSHNIAILLRALNVTADEVCEALIEGNSDTLGPELLEC<br>LLKMAPTKEEEDKLKELKDDDDGSPSKIGPAEKFLKALLNIPFAFKRIDAMLYIV<br>KFESEIEYLNRSFDTLEAATGELKNTRMFLKLLEAVLKTGNRMNIGTNRGDAHAF<br>KLDTLLKLVDIKGADGKTTLLHFVVQEIIKFEGARVPFTPSQSHIGDNMAEQSAF<br>QDDLELKKLGLQVVSGLSSQLINVKKAAAMDSNSLINETAEIARGIAKVKEVITE<br>LKQETGVERFLESMNSFLNKGEKEITELQSHGDNVMKMVKEVTEYFHGNSETHPF<br>RIFAVVRDFLTILDQVCKEVGRVNERTVYGSMPLHSPSNQTATPLFPVVINNNSR<br>LSPSGSLDDDDGSF

No.	Sequence ID	Species	Sequence source/database	Amino acid sequence length (aa)	Protein sequence
10	At-FH18	*Arabidopsis thaliana*	Ref. [27]	1111	MLEDEDYRVYVSRIMSQLREQFPGASFMVFNFRDGDSRSRMESVLTEYDM<br>TIMDY<br>PRHYEGCPLLTMETVHHFLKSAESWLLLSQQNILLSHCELGGWPTLAFML<br>ASLLL<br>YRKQFSGEHRTLEMIYKQAPRELLQLMSPLNPLPSQLRFLQYISRRNVGSQ<br>WPPL<br>DQALTLDCVNLRLIPDFDGEGGCRPIFRIYGQDPFMASDRTSKVLFSMPKRS<br>KAV<br>RQYKQADCELVKIDINCHILGDVVLECITLGSDLEREEMMFRVVFNTAFLR<br>SNIL<br>TLNRGEIDVLWNTTDRFPKDFSAEVIFSEMGAGKKLASVDLPHMEEKDVL<br>PMEAF<br>AKVQEIFSEAEWLDPNSDVAVTVFNQITAANILQESLDSGSPRSPDSRSLLE<br>SAL<br>EKVKEKTKLMISENIVSSPDTSSPEKEKDTMSSHKSYADPNSILKKVDESRG<br>LRV<br>SVQRNVHSKIFSPRMVQSPVTSPLPNRSPTQGSPASISRFHSSPSSLGITSILH<br>D<br>HGSCKDEESTSSSPASPSISFLPTLHPLTSSQPKKASPQCPQSPTPVHSNGPPS<br>A<br>EAAVTSSPLPPLKPLRILSRPPPPPPPPPISSLRSTPSPSSTSNSIATQGPPPPP<br>PPPPLQSHRSALSSSPLPPPLPPKKLLATTNPPPPPPPPLHSNSRMGAPTSSLV<br>L<br>KSPPVPPPPAPAPLSRSHNGNIPPVPGPPLGLKGRGILQNLKGQGQTRKANL<br>KPY<br>HWLKLTRAVQGSLWAEAQKSDEAATAPDFDISELEKLFSAVNLSSDSENN<br>GGKSG<br>RRARPKVEKVQLIELRRAYNCEIMLSKVKIPLPDLMSSVLALDESVIDVDQ<br>VDNL<br>IKFCPTKEEAELLKGFTGNKETLGRCEQFFLELLKVPRVETKLRVFSFKIQF<br>HSQ<br>VTDLRRGLNTIHSAANEVRGSAKLKRIMQTILSLGNALNHGTARGSAIGFR<br>LDSL<br>LKLTDTRSRNSKMTLMHYLCKVLAEKLPELLNFPKDLVSLEAATKIQLKYL<br>AEEM<br>QAISKGLEKVVQEFTASETDGQISKHFRMNLKEFLSVAEGEVRSLASLYST<br>VGGS<br>ADALALYFGEDPARVPFEQVVSTLQNFVRIFVRSHEENCKQVEFEKKRAQ<br>KEAEN<br>EKLKKGVYNEN

No.	Sequence ID	Species	Sequence source/database	Amino acid sequence length (aa)	Protein sequence
11	At-FH11	*Arabidopsis thaliana*	Ref. [27]	884	MVYFRQIFLMIIVVSLHCCKVRFFCIVANAKELDDWKVLTVENGERYRTHVGRYA GEEGGEKIKLRVLEKFRALLDLIKPSTSRRRNLAESASFSPWPAPSPSPFPNGGP IESPAYPPAPPRPIPPHLRRPLPQRTHPLEQPEIQRRKHEKGGTFKKILVPVVAS TASAIGFVVCVVGVFCLCARRKRKMNGKTLSFKRKKGKSQSSTRKVSVNPTLDFL YLNSLGVDLERQNSVSVKEIRETEKDLNGINGGLLEEEVKRSIETEISHDWDNAS SYSTKEIVSVHENDEEQTVNSVSVPVVVINDSSDDDESFHSVGGGSQYSNPRLSN ASSASGSVNVGSSQRFSEHKLDIPECSRSDFGISVSAPPPPPPPPPPLPQFSNKR IHTLSSPETANLQTLSSQLCEKLCASSSKTSFPINVPNSQPRPPPPPPPPQQLQV AGINKTPPPPLSLDFSERRPLGKDGAPLPKLKPLHWDKVRATPDRTMVWDKLRTS SFELDEEMIESLFGYTMQSSTKNEEGKSKTPSPGKHLLEPKRLQNFTILLKALNA TADQICSALGKGEGLCLQQLEALVKMVPTKEEELKLRSYKGAVDELGSAEKFLRA LVGVPFAFQRAEAMLYRETFEDEVVHLRNSFSMLEEACKELKSSRLFLKLLEAVL KTGNRMNVGTIRGGAKAFKLDALLKLSDVKGTDGKTTLLHFVVQEISRSEGIRVS DSIMGRIMNQRSNKNRTPEEKEEDYRRMGLDLVSGLNTELRNVKKTATIDLEGLV TSVSNLRDGLGQLSCLASEKLKGDEENRAFVSSMSSFLRYGEKSLEELREDEKRI MERVGEIAEYFHGDVRGDEKNPLRIFVIVRDFLGMLDHVCRELRCVRVPNSPSPL APFR

No.	Sequence ID	Species	Sequence source/database	Amino acid sequence length (aa)	Protein sequence
12	At-FH10	*Arabidopsis thaliana*	Ref. [27]	841	MDGLCYVIFIIFSLLSCAFSPLSYASPATFSRRHLLQAPVTDPSTFSPPFFPLYS<br>STSPPPPPSPPQPLPPPAPTFATFPANISALVLPRSPKPQTPSRTLLIPAISAVL<br>AAATLIALAFFFYGRWRGQTSHFKDESKSLASDISQSQQTLPCPPPRNNNTQNK<br>LSVAPSTSDVLYLGNVVTSSGSGFVKPESPDISPLPPLPARSFLLQHHSEANLDE<br>EEEDDDFYSPLASIAGQESRDRRINPYSNCSCSISSHSDSPAMSPSAAMSPPMNS<br>TAPHWSTNQNTHSPSSPERTVRNNKRYGGQSLRMFSLWNQNLGFPRISSASTSPE<br>RGMIRTPDAYARSSMYSSVSTTPDRFFRKVLDSSPPRWNDFSRNVKSLFLSSTSA<br>SPARDFCINISESSRSLKSSWEKPELDTTQQRESAAAAVTLPPPQRPPPAMPEPP<br>PLVPPSQSFMVQKSGKKLSFSELPQSCGEGTTDRPKPKLKPLPWDKVRPSSRRTN<br>TWDRLPYNSSNANSKQRSLSCDLPMLNQESKVLDPRKSQNVAVLLTTLKLTTNDV<br>CQALRDGHYDALGVELLESLARVAPSEEEEKKLISYSDDSVIKLAPSERFLKELL<br>NVPFVFKRVDALLSVASFDSKVKHLKRSFSVIQAACEALRNSRMLLRLVGATLEA<br>GMKSGNAHDFKLEALLGLVDIKSSDGRTSILDSVVQKITESEGIKGLQVVRNLSS<br>VLNDAKKSAELDYGVVRMNVSKLYEEVQKISEVLRLCEETGHSEEHQWWKFRESV<br>TRFLETAAEEIKKIEREEGSTLFAVKKITEYFHVDPAKEEAQLLKVFVIVRDFLK<br>ILEGVCKKMEVTSSLA
13	At-FH17	*Arabidopsis thaliana*	Ref. [27]	488	MELLSSIATEGPPPPPPPPLLQPHHSALSSSPLPPPLPPKKLLATTNTPPPPPPP<br>LHSNSQMGAPTSSLVLKSPHNLKGQGQTRKANLKPYHWLKLTRAVQGSLWAEAQK<br>SDEAATAPDFDISEIEKLFSAVNLSSNSENNGGKSGRRARPKVEKVQLIELKRAY<br>NCEIMLSKVKIPLPDLMSSVLALDESVIDVDQVDNLIKFCPTKEEAELLKGFIGN<br>KETLGRCEQFFLELLKVPRVETKLRVFSFKIQFHSQVTDLRRGLNTIHSATNEAS<br>RFFVQIVRGSTKLKRIMQTILSLGNALNHGTARGSAIGFHLDSLLKLTDTRSRNI<br>FIFVLAEKLPGLLNFPKDMVSLEAATNIQLKYLAEEMQATSKGLEKVVQEFTASE<br>TDCQISKHFHMNLKEFLSVAEGEVRSLASLYSTVGGSADALALYFGEDPARVPFE<br>QVVSTLQNFVRIFVRSHEENCKQVEFEKKRAQKEAENEKLKKGVYNEN

No.	Sequence ID	Species	Sequence source/database	Amino acid sequence length (aa)	Protein sequence
14	At-FH1	*Arabidopsis thaliana*	Ref. [27]	1051	MLFFLFFFYLLLSSSSDLVFADRRVLHEPFFPIDSPPPSPPSPPPLPKLPFSSTT<br>PPSSSDPNASPFFPLYPSSPPPPSPASFASFPANISSLIVPHATKSPPNSKKLLI<br>VAISAVSSAALVALLIALLYWRRSKRNQDLNFSDDSKTYTTDSSRRVYPPP<br>PATA<br>PPTRRNAEARSKQRTTTSSTNNNSSEFLYLGTMVNQRGIDEQSLSNNGSSSR<br>KLE<br>SPDLQPLPPLMKRSFRLNPDVGSIGEEDEEDEFYSPRGSQSGREPLNRVGLP<br>GQN<br>PRSVNNDTISCSSSSSGSPGRSTFISISPSMSPKRSEPKPPVISTPEPAELTDYR<br>FVRSPSLSLASLSSGLKNSDEVGLNQIFRSPTVTSLTTSPENNKKENSPLSSTS<br>T<br>SPERRPNDTPEAYLRSPSHSSASTSPYRCFQKSPEVLPAFMSNLRQGLQSQL<br>LSS<br>PSNSHGGQGFLKQLDALRSRSPSSSSSSVCSSPEKASHKSPVTSPKLSSRNSQ<br>SL<br>SSSPDRDFSHSLDVSPRISNISPQILQSRVPPPPPPPPPLPLWGRRSQVTTKAD<br>T<br>ISRPPSLTPPSHPFVIPSENLPVTSSPMETPETVCASEAAEETPKPKLKALHW<br>DK<br>VRASSDREMVWDHLRSSSFKLDEEMIETLFVAKSLNNKPNQSQTTPRCVLP<br>SPNQ<br>ENRVLDPKKAQNIAILLRALNVTIEEVCEALLEGNADTLGTELLESLLKMA<br>PTKE<br>EERKLKAYNDDSPVKLGHAEKFLKAMLDIPFAFKRVDAMLYVANFESEVE<br>YLKKS<br>FETLEAACEELRNSRMFLKLLEAVLKTGNRMNVGTNRGDAHAFKLDTLLK<br>LVDVK<br>GADGKTTLLHFVVQEIIRAEGTRLSGNNTQTDDIKCRKLGLQVVSSLCSELS<br>NVK<br>KAAAMDSEVLSSYVSKLSQGIAKINEAIQVQSTITEESNSQRFSESMKTFLK<br>RAE<br>EEIIRVQAQESVALSLVKEITEYFHGNSAKEEAHPFRIFLVVRDFLGVVDRV<br>CKE<br>VGMINERTMVSSAHKFPVPVNPMMPQPLPGLVGRRQSSSSSSSSSTSSSDED<br>EHN<br>SISLVS

No.	Sequence ID	Species	Sequence source/database	Amino acid sequence length (aa)	Protein sequence
15	At-FH3	*Arabidopsis thaliana*	Ref. [27]	764	MGRLRLAFLAISLVVFVCVSEEIFSRGGLNLLRFSVYGEDVAEQTWIHQNP RRKL ISYPKKFSVSAPNLAFGPAPSFAPGPGPSFAPGPAPNPRSYDWLAPASSPNEP PA ETPDESSPSPSEETPSVVAPSQSVPGPPRPPPQREKKDDILMKLIIAVASTAVL T FVFVALMFLCCFKRNCNNAVGSRDGPRDEGPLLRLSTGSTENSPTVASTSR KMFS VASSKKRSFLSRVSLKRNGHEFSTAESSSAAGLPPLKLPPGRSAPPPPPAAAP PP QPPPPPPPKPQPPPPPKIARPPPAPPKGAAPKRQGNTSSGDASDVDSETGAPK TK LKPFFWDKMANPDQKMVWHEISAGSFQFNEEAMESLFGYNDGNKNKNG QKSTDSS LRESPLQYIQIIDTRKAQNLSILLRALNVTTEEVVDAIKEGNELPVELLQTLL KM APTSEEELKLRLYSGDLHLLGPAERFLKILVDIPFAFKRIESLLFMISLQEEVS G LKEALGTLEVACKKLRNSRLFLKLLEAVLKTGNRMNVGTFRGDAQAFKL DTLLKL SDVKGTDGKTTLLHFVVLEIIRSEGVRALRLQSRSFSSVKTDDSNADSKLED VKR AAIIDADGLAATLANISGSLTNAREFLKTMDEESDFERALAGFIERADADFK WLK EEEERIMVLVKSSADYFHGKSAKNEGLRLFAIVRDFLIMLEKVCREVKETT KTTN HSGKKESEMTTSDSNQPSPDFRQRLFPAIAERRMDSSDDSDDEEDSSPS

No.	Sequence ID	Species	Sequence source/database	Amino acid sequence length (aa)	Protein sequence
16	At-FH13	*Arabidopsis thaliana*	Ref. [27]	1324	MALFRKLFYRKPPDGLLEICDRVFVFDCCFSTDSWEEENYKVYMAGVVNQLQEHF<br>PEASSLVFNFREVGTRSVMADVLSEHGLTIMDYPRHYEGCSLLPVEVMHHFLRSS<br>ESWLSLGPNNLLLMHCESGAWPVLAFMLAALLIYRKQYSGESKTLDMIYKQAPRE<br>LLRLFSPLNPIPSQLRYLQYVSRRNLVSEWPPLDRALTMDCVILRFIPDVSGQGG<br>FRPMFRIYGQDPFFVDDKKPKLLYTTPKKGKHLRVYKQAECELVKIDINCHVQGD<br>IVIECLSLNDDMEREVMMFRVVFNTAFIRSNILMLNRDEVDTLWHIKEFPKGFRV<br>ELLFSDMDAASSVDLMNFSSLEEKDGLPIEVFSKVHEFFNQVDWVDQTDATRNMF<br>QQLAIANAVQEGLDGNSSPRLQGLSPKSIHDIMKHAAIENSAKFKLSSMSEVETI<br>DTPEKPPTDSVKKFIAEDVHSVLQINNQEQNASEDATKLLHQESPSLKLVHHSAT<br>VKPLVDDSKSPENAEENFPKSPSAHDGKAISFSPPTPSPPHPVRPQLAQAGAPPP<br>PPPLPAAASKPSEQLQHSVVQATEPLSQGNSWMSLAGSTFQTVPNEKNLITLPPT<br>PPLASTSHASPEPSSKTTNSLLLSPQASPATPTNPSKTVSVDFFGAATSPHLGAS<br>DNVASNLGQPARSPPPISNSDKKPALPRPPPPPPPPPMQHSTVTKVPPPPPPAPP<br>APPTPIVHTSSPPPPPPPPPPPAPPTPQSNGISAMKSSPPAPPAPPRLPTHSASP<br>PPPTAPPPPPLGQTRAPSAPPPPPPKLGTKLSPSGPNVPPTPALPTGPLSSGKGR<br>MLRVNLKNSPAKKLKPYHWLKLTRAVNGSLWAETQMSSEASKYALFILLSLISLM<br>PPDSCMISNSLILYLLVRAPDIDMTELESLFSASAPEQAGKSRLDSSRGPKPEKV<br>QLIEHRRAYNCEIMLSKVKVPLQDLTNSVLNLEESALDADQVENLIKFCPTREEM<br>ELLKGYTGDKDKLGKCELFFLEMMKVPRVETKLRVFSFKMQFTSQISELRNSLGV<br>VNSAAEQVKNSEKFKRIMQTILSLGNALNQGTARGAAVGFKLDSLPKLSETRARN<br>NRMTLMHYLCKVSFYSLRFCSFVDVLEEERYSLMDSLQILAEKIPEVLDFTKELS<br>SLEPATKIQLKFLAEEMQAINKGLEKVVQELSLSENDGPISHNFNKILKEFLHYA<br>EAEVRSLASLYSGVGRNVDGLILYFGEDPAKCPFEQVVSTLLNFVRLFNRAHEEN<br>GKQLEAEAKKNAAEKEKPKTGGLDTEIKKPLNEEVKEEKTKTSGLGKEMSDRLKE<br>RTAP

No.	Sequence ID	Species	Sequence source/database	Amino acid sequence length (aa)	Protein sequence
17	At-FH5	*Arabidopsis thaliana*	Ref. [27]	900	MVGMIRGGMGDQNWSRLVFWLILFSGLLVITLEENPEKDEIFLSQFMAPST<br>GQVN<br>EHMEETSWAQRCWQDSDCVKEAVAEFNLCFPGSKDSRELFGLNHTNLKQ<br>TLLDCI<br>QEKGKLNGHNPKYLELLSSMLDIPRRNLATKPGSSPSPSPSRPPKRSRGPPR<br>PPT<br>RPKSPPPRKSSFPPSRSPPPPPAKKNASKNSTSAPVSPAKKKEDHEKTIIIAVV<br>V<br>TAVSTFLLAALFFLCCSRVCGNGSGGRKNDERPLLSLSSSDYSVGSSINYGG<br>SVK<br>GDKQGHQSFNIYSNQGKMSSFDGSNSDTSDSLEERLSHEGLRNNSITNHGL<br>PPLK<br>PPPGRTASVLSGKSFSGKVEPLPPEPPKFLKVSSKKASAPPPPVPAPQMPSSA<br>GP<br>PRPPPPAPPPGSGGPKPPPPPGPKGPRPPPPMSLGPKAPRPPSGPADALDDDA<br>PK<br>TKLKPFFWDKVQANPEHSMVWNDIRSGSFQFNEEMIESLFGYAAADKNKN<br>DKKGS<br>SGQAALPQFVQILEPKKGQNLSILLRALNATTEEVCDALREGNELPVEFIQT<br>LLK<br>MAPTPEEELKLRLYCGEIAQLGSAERFLKAVVDIPFAFKRLEALLFMCTLHE<br>EMA<br>FVKESFQKLEVACKELRGSRLFLKLLEAVLKTGNRMNDGTFRGGAQAFKL<br>DTLLK<br>LADVKGTDGKTTLLHFVVQEIIRTEGVRAARTIRESQSFSSVKTEDLLVEET<br>SEE<br>SEENYRNLGLEKVSGLSSELEHVKKSANIDADGLTGTVLKMGHALSKARD<br>FVNSE<br>MKSSGEESGFREALEDFIQNAEGSIMSILEEEKRIMALVKSTGDYFHGKAG<br>KDEG<br>LRLFVIVRDFLIILDKSCKEVREARGRPVRMARKQGSTASASSETPRQTPSL<br>DPR<br>QKLFPAITERRVDQSSSDSD

No.	Sequence ID	Species	Sequence source/database	Amino acid sequence length (aa)	Protein sequence
18	At-FH15	*Arabidopsis thaliana*	Ref. [27]	815	MSLVEISGSDAMAAPMPGRVPPPPPRPPPMPRRLPPMFDAFDHTGAGMVW<br>GFPRP<br>AKKRASLKPLHWVKITSDLQGSLWDELQRRHGDSQTAIELDISELETLFFV<br>EAKP<br>EKIRLHDLRRASYRVFNVRSYYMRANNKVINLSMPLPDMMTAVLAMDES<br>VVDVDQ<br>IEKLIKFCPTNEEMELLKTYTGDKAALGKYEQYLLELMKVPRLEAKLRVFS<br>FKTQ<br>FGTKITELKERLNVVTSACEEVRSSEKLKEIMKKIPCLGNTSNQGPDRGKSS<br>VVD<br>KNLSFSSGIQLKEIMKKIPCLGNTSKSNPRVGVKLDSSVSDTHTVKSMHYY<br>CKVL<br>ASEASELLDVYKDLQSLESASKIQVKSLAQNIQAIIKRLEKLKQELTASETD<br>GPA<br>SEVFCNTLKDFISIAETEMATVLSLYSVVRKKADALPPYFGEDPNQCPFEQL<br>TMT<br>LFNFIKLFKKAHEENVKQADLEKKKAMKQIDLRRANDTEIMLTKVNIPLAD<br>MMAA<br>VLGMDEYVLDVDQIENLIRFCPTKEEMELLKNYTGDKATLGKCEQLAKAK<br>APLKE<br>HFRVINAFPSLTPQYFLEVMKVPGVESKLRAFSFKIQFGTQIAELNKGLNAV<br>NSA<br>CEEVRTSEKLKEIMANILCMGNILNQGTAEGSAVGFKLKSLLILSDTCAPNS<br>KMT<br>LMHYLCKVLASKASDLLDFHKDLESLESASKIQLKSLAEEIQAITKGLEKLN<br>KQL<br>TASESDGPVSQVFRKVLKDFISMAETQVATVSSLYSSVGKNADALAHYFG<br>EDPNH<br>YPFEKVTTTLLSFIRLFKKAHEENVKQADLDKNKDAKEAEMEKTK

No.	Sequence ID	Species	Sequence source/database	Amino acid sequence length (aa)	Protein sequence
19	At-FH21	*Arabidopsis thaliana*	Ref. [27]	853	MSPVEISGADAVVSPPMRGRVPLPPPPPPPPPMRRRAPLPPPPPPPMRRRAP<br>LP<br>PPPPPAMRRRVLPRPPPPPPPLPMFDAEVLCCCYPPTRVRREAPLPPPPLIFV<br>GA<br>PPPTCALKGIVCCFPCPSKKKSSLKRFNWVKITRALPGSLWDELQIQQVCH<br>GDIE<br>DEQILCAIELDVSEIETFFSLGAAKPEKDPLIDLRRATDTELTLMLLNIRLPA<br>DM<br>MAAIMAMDESVLDDDEIRGLINLFPTKENMELLMSYTGGKWTLEKWEQY<br>FQELRK<br>VLRVESKLRVFYFKIQFSTKITQFKKRLNVVNSACEEVCSSQKLKEIMKKIT<br>CLG<br>NTSNQGTGRGVTVGFNLDSLCVKSMHNFCKVLASEASDLLDVHKDLQSLE<br>SASKK<br>QLKSLAEEMQDIIRDLEKLNQELTAAETDGPDSQVFRNTLKDFISIAETEVK<br>TVL<br>SLYSVVGKNAVALVNYFGEDPKWCPFEQVTATLLHFIRLFEKAHEENVKK<br>ADLEK<br>KKAANETEMKHVVHNSWLNNLNIRASTLIDQRRAFNTMIMLQKVEMPLP<br>DMMAAV<br>LGMDESVLDVDQIENLIRFCPTKEEMKLLKNYTGDKATLGKCEQYFLELM<br>KVPGV<br>ESKLRVFSFKIHFGTQIKELNKGLNTVNSACEEIRTSQKLKEIMKIILCLGNIL<br>N<br>QGTARGSAVGFKLDSLLNLSEKCSANTNMTLMHYLCKVLASKASDLLDFH<br>KDLEN<br>LESASKIHLKSLAEEMVAITKGLQKLNQELTASESDGPISEVFRKLLKDFISV<br>AE<br>TQVATVSSLYSSVGGNTDALVHYFGEDPNDYPFEQAAHQENVKQAELEK<br>KKAAKE<br>AEMEKTKKRVSLTNKKASGVGEEESCLI

No.	Sequence ID	Species	Sequence source/database	Amino acid sequence length (aa)	Protein sequence
20	At-FH20	*Arabidopsis thaliana*	Ref. [27]	1649	MALFRRFFYKKPPDRLLEISERVYVFDCCFSSDVMGEDEYKVYLGGIVAQL QDHF PEASFMVFNFREGEQRSQISDVLSQYDMTVMDYPRQYESCPLLPLEMIHHF LRSS ESWLSLEGQQNVLLMHCERGGWPVLAFMLSGLLLYRKQYHGEQKTLEM VHKQAPK ELLHLLSPLNPQPSQLRYLQYISRRNLGSDWPPSDTPLLLDCLILRDLPHFEG KK GCRPILRVYGQDPKARTNRSSILLFSTLKTKKHTRLYQQEECILVKLDIQCR VQG DVVLECIHLHDDLVSEEMVFRIMFHTAFVRANILMLQRDEMDILWDVKDQ FPKEF KAEVLFSGADAVVPPITTSTLSDDENDFDMTSPEEFFEVEEIFSDVIDGPDHK RD SDSFVVVDTASDDSEGKEVWKGDVEPNAFLDCASDDSNHKHDMHAETST DPVKDI TVDDVQYRSDGKADSNIDSVKDIGIDDGDEQRKRRTVEAKENDSSTVQTQ SKGDE ESNDLESMSQKTNTSLNKPISEKPQATLRKQVGANAKPAAAGDSLKPKSK QQETQ GPNVRMAKPNAVSRWIPSNKGSYKDSMHVAYPPTRINSAPASITTSLKDGK RATS PDGVIPKDAKTKYLRASVSSPDMRSRAPICSSPDSSPKETPSSLPPASPHQAP PP LPSLTSEAKTVLHSSQAVASPPPPPPPPLPTYSHYQTSQLPPPPPPPPPFSSER PNSGTVLPPPPPPPPPFSSERPNSGTVLPPPPPPPLPFSSERPNSGTVLPPPPSP PWKSVYASALAIPAICSTSQAPTSSPTPPPPPPAYYSVGQKSSDLQTSQLPSP PP PPPPPPFASVRRNSETLLPPPPPPPPPPFASVRRNSETLLPPPPPPPPWKSLYAS TFETHEACSTSSSPPPPPPPPPFSPLNTTKANDYILPPPPLPYTSIAPSPSVKIL PLHGISSAPSPPVKTAPPPPPPPPFSNAHSVLSPPPPSYGSPPPPPPPPPSYGSP PPPPPPPPPSYGSPPPPPPPPGYGSPPPPPPPPPSYGSPPPPPPPPFSHVSSIPP PPPPPPMHGGAPPPPPPPPMHGGAPPPPPPPPMHGGAPPPPPPPPMHGGAPPP PP PPMFGGAQPPPPPPPMRGGAPPPPPPPPMRGGAPPPPPPPPMRGGAPPPPPPPPMH GGA PPPPPPPMRGGAPPPPPPPGGRGPGAPPPPPPPGGRAPGPPPPPGPRPPGGGPP P PPMLGARGAAVDPRGAGRGRGLPRPGFGSAAQKKSSLKPLHWVKVTRAL QGSLWD ELQRHGESQTPSEFDVSEIETLFSATVQKPADKSGSRRKSVGAKPEKVQLID LRR ANNTEIMLTKVKMPLPDMMAAVLAMDESVLDVDQIENLIKFCPTKEEMEL LKNYT GDKTTLGKCEQYFLELMKVPRVEAKLRVFSFKFQFGTQITEFKKSLNAVNS ACEE VRSSQKLKEIMKKILYLGNTLNQGTARGAAVGFKLDSLSKLSDTRAANSK MTLMH YLCKVLASKASVLLDFPKDLESLESASKIQLKSLAEEMQAIIKGLEKLNQEL TAS ESDGPVSDVFRKTLGDFISVAETEVATVSSLYSVVGRNADALAHYFGEDPN RCPF EQVTATLLNFIRLFKKAHEENVKQAELEKKKALKEAEMEKAKGVNLTKKP

No.	Sequence ID	Species	Sequence source/database	Amino acid sequence length (aa)	Protein sequence
21	At-FH19	*Arabidopsis thaliana*	Ref. [27]	464	MSLVDISGAYSLVPLPPPPPPLMRRRAPLPPPPPPPLMRRRAPPPPPPPLMRR<br>RA<br>PPPPPPPPLPRPCSRPPKTKCSLKPLHWVKKTRALPGSLWDELQRRQECRDI<br>EDE<br>QILCAIELSVSEIETIFSLGAKPKPKPEPEKVPLIDLRRATNTEIRLMLLNIRLP<br>DMIAAAMAMDESRLDDFDQIENLINLFPTKEDMKFLLTYTGDKGNCEQLF<br>QYLQE<br>VVKVPRVESKLRVFSFKIQFGTQITKLTKGLNAVNSACEEIRTSQKLKDIME<br>NIL<br>CLGNILNQGTGRGRAVGFRLDSLLILSETRADNSKMTLMHYLCKVLASKA<br>SDLLD<br>FHKDLESLESASKIQLKSLAEEIQAITKGLEKLKQELTASETDGPVSQVFRK<br>LLK<br>EFISNAETQVATVMALYYPARGNAEALAHYFGYHYPFEQVTATLLSFIRLF<br>KKAH<br>EENVKQAELEKKKAAKEAEMEKTK
22	At-FH16	*Arabidopsis thaliana*	Ref. [27]	722	MSPVEITGADAVVTPPMRGRVPLPPPPPPPMRRSAPSPPPMSGRVPPPPPPPP<br>MF<br>DPKGAGRVICCLRPGQNKSSLKRFQCGKLTNAWEELQRHGEAQTAPEFDL<br>SEIEA<br>LFSAAVQNQADKSGSRREAFEANPDKLQLISGADALVPLPPPPPPMPRRSPP<br>PPP<br>PRFDAFDHKGARMVCGFRCPVTKRSSLKPLHWVKITRALQGSLWDELQIQ<br>YGESQ<br>TAIELDVPEIETLFSVGAKPRPKPKPEKVPLIDLKRANNTIVNLKILKMPLPD<br>MM<br>AAVMAMDESVLDVDQIENLIQLCPTKEEMELLKNYTGDKATLGKSEQCLL<br>ELMKV<br>PRFEAKLRVLSFKIPFGTKITKFRKMLNVVNSACEEVRSSQMLKEIMKIILFL<br>GN<br>TLNQGTARGSAVGFRLDSLLILSETRADNNKMTLMHYLCKVLASKAADLL<br>DFHKD<br>LQSLESTLEINLKSLAEEIHAITKGLEKLKQELTASETDGPVSQVFRKLLKDF<br>IS<br>SAETQVATVSTLYSSARINADALAHYFGEDPNHYPFEKVSATLLSFIRLFKK<br>AHQ<br>ENVKQEDLEKKKAATEDVFGGPDHNIDSDTSLDDSEAKSPSRIRPPPSIPRPP<br>SR<br>PRYACCRIPAVNPPPRLVCGPYPLPRLVRVGSPSPPPPSMSGGAPPPPPPPPM<br>LV<br>ASRTAPPPHLSHVRSIPFQTRLVMGTSPLPLLVREGAPPPTLPSMSGGAPPPP<br>PP<br>LPMLRYQ

No.	Sequence ID	Species	Sequence source/database	Amino acid sequence length (aa)	Protein sequence
23	At-FH9	*Arabidopsis thaliana*	Ref. [27]	782	MQNFWFAIFFFLLTCAPPSPLSYASTVTLSRRLLYDYESPLPLPLSPISPPFFP<br>L<br>ESSPPSPPPPLPPTPPTTFAVFPTFPANISALVLPRSSKPHHTSPTLLLPALSAV<br>LVIATVIGLALFLYGRHRGQTRHLKNSHCSSSNTSSYGDEQSHITTNFNMA<br>ATTS<br>PSEVFYLNTEESDHIRTGGTFFLKQDSPEIRPLPPLPPRSFHHNNYETEVNEE<br>DE<br>EEEEDVFFSPMASLPGSANSSPSHSCSSSCSGWVSPARSFSITMSPPNPRYSD<br>AT<br>NLQSPSPERLRVRKNYNGNGSSSLRMFSFWNQNMGFGFPRISSASTSPDRG<br>FIRT<br>PLSSLYSSVSTSPDGLFRKFLDSSPPIWNDFSRNVKSVLLSHTASSRRDFVINI<br>G<br>ESSSQQSKVPALPPPTRPPPLVPPSQPFVVQNDVKKQSFSDQPPKQLHWERL<br>RSS<br>SSKLSKEMVETMFIANSSNPRDLPIQNQVLDPRKAQNIATLLQLLNLSTKDV<br>CQA<br>LLDGDCDVLGAELLECLSRLAPSKEEERKLKSFSDGSEIGPAERFLKELLHV<br>PFV<br>FKRVDALLFVANFHSEIKRLRKSFSVVQVACEELRNSRMFSILLEAILKTGN<br>MMS<br>VRTNRCGDADAFKLDTLLKLVDVKGLDGRSSLLHFVVQEMMKSEGSVRA<br>LEGIRN<br>LNTELSNVKKSADIEYGVLRSNVSRICQGLKNIEALLLLSEESGSYGDQWL<br>KFKE<br>RMTRFLKTAAEEIVKIKIRESSTLSALEEVTEQFHGDASKEGHTMRIFMIVR<br>DFL<br>SVLDQVCKEMGD

No.	Sequence ID	Species	Sequence source/database	Amino acid sequence length (aa)	Protein sequence
24	At-FH6	*Arabidopsis thaliana*	Ref. [27]	899	MKALQSRFFFFFFFYIFFSVSVSSEAHRRILHQPLFPESSTPPPPDFQSTPSPPL<br>PDTPDQPFFPENPSTPQQTLFPPPPPPVSADVNGGLPIPTATTQSAKPGKKVA<br>IV<br>ISVGIVTLGMLSALAFFLYRHKAKHASDTQKLVTGGGDGGGSRRFQEDSG<br>PPTTT<br>SSTFLYMGTVEPTRVSASESNGGTNGPVNSSPYRKLNSAKRSERYRPSPEL<br>QPLP<br>PLAKPPQPSDNSPSALSPSSSSSGEECRDTAFYTPHGSAISSDDGYYTAFPRS<br>AN<br>GSLPHSKRTSPRSKFGSAPTTAASRSPEMKHVIIPSIKQKLPPPVQPPPLRGLE<br>S<br>DEQELPYSQNKPKFSQPPPPPNRAAFQAITQEKSPVPPPRRSPPPLQTPPPPPP<br>P<br>PPLAPPPPPQKRPRDFQMLRKVTNSEATTNSTTSPSRKQAFKTPSPKTKAVE<br>EVN<br>SVSAGSLEKSGDGDTDPSKPKLKPLHWDKVRASSDRATVWDQLKSSSFQL<br>NEDRM<br>EHLFGCNSGSSAPKEPVRRSVIPLAENENRVLDPKKSQNIAILLRALNVTRE<br>EVS<br>EALTDGNPESLGAELLETLVKMAPTKEEEIKLREYSGDVSKLGTAERFLKTI<br>LDI<br>PFAFKRVEAMLYRANFDAEVKYLRNSFQTLEEASLELKASRLFLKLLEAVL<br>MTGN<br>RMNVGTNRGDAIAFKLDTLLKLVDIKGVDGKTTLLHFVVQEITRSEGTTTT<br>KDET<br>ILHGNNDGFRKQGLQVVAGLSRDLVNVKKSAGMDFDVLSSYVTKLEMGL<br>DKLRSF<br>LKTETTQGRFFDSMKTFLKEAEEEIRKIKGGERKALSMVKEVTEYFHGNAA<br>REEA<br>HPLRIFMVVRDFLGVLDNVCKEVKTMQEMSTSMGSASARSFRISATASLPV<br>LHRY<br>KARQDDTSSDSEHSSNSST

No.	Sequence ID	Species	Sequence source/database	Amino acid sequence length (aa)	Protein sequence
25	Gl-FH1	*Gracilariopsis lemaneiformis*	NCBI protein database	1015	MQAIVEPTYAQGNRNVDFMDEIRQKNAEREKRIQEQAAFEGNESQRKAKL<br>LEQVF<br>GCEVGVEEVDEFVEVFMRYNEATSEQQGDKVDRQRTAEELLSKTFTGRSH<br>SAHRA<br>MEASSEEDGFSDIAKFIHEEELDEEELGDISLLKDKLAQKRSSDSEDSDRTE<br>DIE<br>EDRENRDADVLREAVQVLLKGVKKTGRADRFRADDVLKNADSLNNGDV<br>NENEIIS<br>QITGALAALVNESRAPENEGSGRKFFSAQELVDKVKVMRHETDPSTSGEAS<br>ASGV<br>PHPPPIPGASVVHEHGKAPTGPPLPPGIGGAAPSGGAPPPPPPPPPPPRRPLTRP<br>S<br>GAAAPPPPPPPPSRSRPPSSSSGGAGPPPPPPPPPPPLRSRPPSSPSGGAGPPPPP<br>PPPPPRSRVQRPSPGDAPPPPPPPPPPPGSKSAIPPPPPIGGSKKQGIPPLPPLL<br>AGLKKSSGPPPPPPPVSGLKKGSAAPLPPPMPGLRKPRGPPKAPPKGGLKTP<br>ADS<br>TAPKAPVPGSSTPKSQVQKRNDTKRLNWATISNVKVSKTLYAKEEFQNIVA<br>LDED<br>TEKDLLEIFSNRPQPRLQNANEEQKTEKQGGSHAAGILEQRKMQNTLIMLR<br>KFKV<br>SPKQITEAVRSLDPLSTQLSLDNVSALISNAFKPEELEMAKNFAAPEEEVAK<br>LNP<br>AESLAYHVARVPRWTAKVKAMMTMRTAEEVEDEIRVSLTDVISASKEVL<br>NSDRLQ<br>KILAMVLAIGNFLNAGTAKGSARGFKLEALTKLSEIKSREKGTSLLHYIADL<br>AER<br>KSPDTLLFAEDMPHVVKARRVVKEDIKRELTTFQRAVAMLGKELTEIVKE<br>EQAKG<br>GGQNVSLPSTPPPPKSSRRLSNLSDSQSTGSGDPSKENGDSKKQPTNDVAD<br>GNDT<br>AGEGNDESTQDSRNPLEVAKETYTKAEAAIEDLLKLQEEMLRTFMELAVL<br>LGEDS<br>KSVKVEDLFMTLWTFMEAFGQAVKDNKKRAEENARKEKRAKREAEDRK<br>LREEHEK<br>IKQSAASKVASEDGNVSIPTGVRSD

No.	Sequence ID	Species	Sequence source/database	Amino acid sequence length (aa)	Protein sequence
26	Gl-FH2	*Gracilariopsis lemaneiformis*	NCBI protein database	639	MSKKYDARSTLEDRLGPDVLARLQSMAHEFVRFERGQFGFYIDPARYAVKQRQVG PLGRGRRRKVVAGVDKDGNIITAWIGVDSRANGARTQGGEMVTIPDEYAEGALGG GGGGGYGPGGGGGYGPGGGGGGPGAGFGPGGGGGPGAGPGAGGGPGAGGPGRGPG GGGGGNAPGAAGAAAAAAAARKNQAANAATPRAPGVPGAPGGAGVPAVPKAPVGQ VAGKDNTARGRQKKPLHWAKVPMNLVNQSFWPTVDESNVRLDEEEIDDLFGVDVV ERFQLAAAEVKPEVLPHKRKHNVNILLANLKMSAEDIKDVVRVPTYKELEAGTLQ ALLLVCPTQEEEMLLAQNANIRNEVDKTDAFMMDLADLKGLRGKILCALAAKTFN DEAVEVIRNMDTFAMIPVEVMNSEKLVRILESILALGNFLNSGTGRGGAHGFKLE ALAMLSTVKDAKGNMLLDYLIFMLERDNPGLLPLDDMPTLARSKELSLEAIGEDV QALLESVTNVSEQIKQIGEDQTLAAFKAEMEVFAEEATKVREEIVSLRGLMMQKL QSMMQHFGERNKMSRGRQEDILRMLREFASDIDTGLVRAKERSERARKQAAKNEA RKAGSAVPPAPPAPPAVEVGGAVDGGGAHAGAQE
27	Gl-FH3	*Gracilariopsis lemaneiformis*	NCBI protein database	593	MARRSKRNDAQVTLEDRLGPDVLKKLETMAGEFVRFEKGDFGFFIDPARYAVKQR QVGPLGRGRRRKVVAGVDKDGNIITAWIGVDNKSKGTKTDAGELVNIPDGYGDGT GHVGTAKPPASAAEVAKQAAEKRAIAGVSSTPEKKDEDGTKPAAPTGPPVPKPPV GNLQAKANEARGRKKKPLHWAKVPKNLLKDAMWTSLDDSSIRLNEDEIDELFGVD VAPQFVAEAEAAKPEVLPHKRKHNVNIILANLKMTTDQIKDVVRVPTYKELEASN LQALLMICPTPEEEQLLNQNQNIIDEVDKTDRFMMELAQLPGLRGKVLCGLSAKT FNEEAIDLIKSMDTYAMIPVEVENSAKLNKMLEIVLALGNFLNSGTGRGGAHGFK LEALAMLSTVKDSRGDTLLDYLVKIIMQDYPHLYPLDDMPTLQKSEALSLDAIGE DVQAILDSVTNVAEQIKQIGDDPSMERFKSEMSAFSEEATKVREEIVSLRALMME KLQHMMAHFGERNKAARGRQEDILRMLREFSADLTAAVARYNERCEREAKKAAKA GGKPPAKPVPSGENVPALPDAPQEGPSVTEKIVSGSRASGTAE

No.	Sequence ID	Species	Sequence source/database	Amino acid sequence length (aa)	Protein sequence
28	Cp-FH1	*Chloropicon primus*	NCBI protein database	1194	MNSIRLNRKLVSFEAFSSELAERVHRPLLRSKRGKHLSSHDSTKLHIASEVL<br>AHV<br>LQHLNESKGASVSSVFGAVKEQKLRELYVLFLKEFRDNGLFDDRNQREGK<br>FTKVW<br>GASFSEKGLSYSLPLVQKLLGELEDPLMTRVEYTAFLTAVRIPESKSRHFVL<br>RLL<br>LDKLAAQKLEVLRLLVAVLQRVSLEEEGTDEEEVREVIEKCGRVIFQSTEV<br>NSDR<br>TIINKSDILAAETCLKMLLEEGQIMLQEGDRATKSRQRLTESTRADPPRRPD<br>PPS<br>RLRRRTSFWGNPKSSSYSAMHMKRVLLHKADAQANSERAARRPARVSSS<br>AEKQAT<br>GTGKENLGSPPYGSGDGASFEISEANLGKIRRYFLKTPTTLISKTRSQEKRTG<br>TA<br>KALAHSLKGLSPRMGSPGQCDPKRALKALSSSPGGARGSPSSPTGLEGDSQ<br>TALN<br>VIDPKEYSKYLVGLPKEVLDKIKHLSATAVARSCESSPGSSTCTDLSGMLEK<br>IDQ<br>LMSSPEGDETSSGRSSLSPGRLKSMLAELTNSPEASASSLSGKKGDSDSKQR<br>GSG<br>GGSPGAVAAKNASPGTRAKAQPGKPGDGATAPPPPPPPPPLPAGKSSAPPP<br>PPP<br>PPPPPPGAKGNIPPPPPPPGGWKNGGPPPPPPPPGGWKNGGPPPPPPPPGAG<br>NKG<br>LARPNPVVKAVKKMKALHWQKLHKTKTKKTVWETEREELDLDVQELESL<br>FSLADK<br>PEKKLEKKKSTKVHLVGLKRSHNISIELSGMKLDFSKIRDSLLDMDDSKFTL<br>EQL<br>HVLKRSVPTDQEATKLRNYTGDKSKLGQVELYFMEVMSIPRLENRIDSLIY<br>KKTF<br>VSCVDKLKEELNVLKQASSQVLSSNSLKLILEGVLAVGNYLNTGTARGSA<br>AGFKI<br>ESLLKLKDIKGKDRKTSLLHFIVKELVKKNDKVRFFTAEIDKVDTASKFQK<br>DYVS<br>LTLQQLSSQQSKLKEEILHSSVVLDETSDLNDRFRDVMVPFAEDSEETLTC<br>MKSI<br>LDDTMGNLAKMRDFLGEDEATASTETFKTLKEFMLIYDSVVNDIRQVEEA<br>EEQKK<br>RAELRAAARRERSKSVCVLSPPTPLGGSDDGDKIADKTSTAPGEGKDLGAA<br>TKAR<br>ARSMSLCSSSAPFVVPAQPPSGVREGKPEALASVNEEEEGEEGASSGSATA<br>TPSK<br>PPSARDEDIAFFSPESSLYSGAESEDEDEMIEEAFAGL*

No.	Sequence ID	Species	Sequence source/database	Amino acid sequence length (aa)	Protein sequence
29	Cp-FH2	*Chloropicon primus*	NCBI protein database	1069	MSANRRKTRTGSVFGALFGAATPQQQLPDRVVLKTPEQGSGNGYPPSAGS<br>RQTMS<br>PQRKSRNSNAMDGSVTPVTPLQLLRLNYTADLPLQSLEEQYARALDFITKH<br>PKAF<br>TELQLSSLLGWLEIAKQSDASSTPRSGGPPTPSASGTPVTPLPSGTPPQMASS<br>NC<br>NPSTSLAAKSLLLLELTKIEPDWAAWQTPRPQSPSKKGDFVSPEGLICELHG<br>KPF<br>NVFLLRGLRDGLNYNSAAWLRLFVSLQGIPLVTKIFLCAIKSLEGKVHTEE<br>GIMD<br>RSDLLGSPNGFEFHSKPSGKEDVLKFNPSNDRCNTEAWHEVMNCTRGLTN<br>NHVCM<br>DGLIANQDSISRLLRALSPVFPDASKLIFEIATALCLYSNEAFNKVMKAMLN<br>VSD<br>SIEVNTNGMCTPKLHAKFAEFTPEQEAPGKSLELPLITLSLVNLLKITDADID<br>LK<br>SHIMTFINIVLTSPMAEGSQADSSKVDSTDLRKAWLEKMLECGLLNAIAEFS<br>DDE<br>DNYLSHTIDIFKDSLSRCLAPTPKKKPVPSGAPPPPPPPGGRGGPPPPPPPPG<br>MP<br>GRPGAPPPPPMPGRAGALVDPGPKPGKKMKAFHWTKLPDTRAKQTFWDS<br>SKNPTQ<br>KKKALDDLRKTLELTSTFESSFSMAVRKKINLNRTKEAKKTVMAMDMKR<br>ATAVGI<br>SLAKFNVPQDTIIRGIIRLDGDIFMSSENVEAIIKCIPTDDEKKVLLSLHAAGN<br>S<br>SKLSDAEKFCLKLLEIPNIDSRMKTFLLKFTLIDQLEDLKNIVQAHTMAVHE<br>IKM<br>SRTFLLLLRSTLVLGNYMNHSTRLGSALGYRMSALSKLKDIRSTDNSNSNL<br>LHYI<br>VKMILKARPDSKSLADEVPHVISKEIKTSLQDVASHLETIESNLMDAKYELD<br>SPV<br>ETVKVEFNVNPEGGGSVDRVAVEIVADAYYDVMTTFVEESTVVKNDLLE<br>AVEKCK<br>KSFSDMVSYFGENPAALQNESEFWDIIASVTLAYKDVHNTITKQMKEEKEK<br>EVWR<br>QKRAKSKSIAAMGSGGVSYKPAEYKRTGYNCPIHDEPVRYERWEKPPEEQ<br>GSEEP<br>SQVQGSKAPAGQDLPSTNGTAEV*

No.	Sequence ID	Species	Sequence source/database	Amino acid sequence length (aa)	Protein sequence
30	Cp-FH3	*Chloropicon primus*	NCBI protein database	962	MGAAGSKLSEGEGGQGVWEVVGAVQPTASRPEYISDRLLAFAYEDVRLG VPSLER VLTSGNVQSASDGYVDCLTHAAQFLQEHHSGSYMMVNLGGLLLEPKLYS LFGNSC VEFVVPWNVPIGCGVAPMDILFAACSSIHNWLSMDDDHVAVLHTRTAPGG HAENF LRFVAACFLTYSLDFDHVSEALDVVMPDKARTNSQQEMRRGSLGSIVRSPL SITM NSPLKKKWLKNKNVEDDKSLKERPSASQRRYGQYFMNVLHSPVLPSWQR TPVMLK RVVLSFEPFGGACGAEHFSVGSHSTSGMGGEPVLVVHRRGQEIWAGVADT GDIMS KRDLVGFEPDLLVVGDIVLSLWAGDRQRQHEMPILSVPLNTAFVDRSIMRL TGRQ VEQSVNFPLPDDFFIDITFDEVNKEALERHSSLPQDALMTMDEARHGWKSV LAAT SGMSVNDFYAYDDVVHEMQARHALSQGNSEAGDRADSNLREALKAVQT GMTVMNT SPGKRHAEDDAESPRLARRRSSLDEETTVLVQQAETRAPPDPPVPPPPPAGG SMP PPPPPPPPGGSGIPPPPPPPGVPGAPPPPPPPGGIPGRARKAYTGPKLRSWYW QT VNNTQGTIWHSVNQNPLIAGKEADSVRSELIKLFPAKASGKQMKMGGAKS KSASV VKYISLARANNISIMLTQFRGHHIDLKTSIMTGQDFSLEQLGVLLQLIPTDDE LR IMKSIPKNELADLSEPESFLAMLSEIPRLRSKIQCRVFMRQFDSWVQEFQDG LDA NLKACNAIRSSKNLKTVLGVSLEVGNLLHVGTSRQGAKGVRLESMLKMR DLRVTK SAGDSGKARNLLDFVIQEVAKVRGGKEPLRESLQAVSKACNYAQSDLLAL MNQLE AGLTLVSEELKQSKDELGWLESFQAKAREVKSGCEHQAEKVLQEAGQIVT YMGEA RNVSSEEVFRLLWNFVLQYDQTLRRM*

No.	Sequence ID	Species	Sequence source/database	Amino acid sequence length (aa)	Protein sequence
31	Cp-FH4	*Chloropicon primus*	NCBI protein database	890	MSRRYERRHSAFSGAPSYGSIAAEAARMVHNRRRHEGKLLLRPVSYQSTN<br>YYSKI<br>SSWSYLGSDDRDEEEEGDHQGTRRVGSEGTGSQKRKKPSPARLLARMML<br>DSSESE<br>SDTESEDVKEVESKAVEEVELKTVVTTEAVFSTPKTTIAATSSEMPRTVTPS<br>APA<br>GTAVTPAPREEAAKEEEEDFSVKPAVLVSENKAEREIVDLSDMTPRRKPRK<br>RGGK<br>TPGKAPGKTPGRTPGRTPGKTPGKTPGRTPGRTPGRKVLGDISNTCTPVKSS<br>ARK<br>NRRHRGTPSAASKSATKGKGGSTTQEQAQGDDHKNHAVSVTLMPPPAPPP<br>PPPPP<br>PGAIAPPPPPPPPPGAGAPPPPPPPPNGAAPPPPPLPLLGKAFQLGTLQKPVVP<br>R<br>RRRKWHWETLPDYKAADTIWREHLYSKRSVTIDSEMLESLFTREISPVKKR<br>STKS<br>RLGPGMKPSKMESQLDQVAGKGGKGGDGGSMAIVALLDVKKASNIEITLA<br>RLTRE<br>SGKEPEEIVEAINQLDARSLTESSTVVECEDGSGGSSEWSRDSLEDTAEAVD<br>RLL<br>KVLPSNEECEGISLWYSDKIKSGDAFDPKVQFGIAERFCLATQQTVGSASRL<br>VSK<br>LRATRFKLEFDIVQDLVSDKMNLMTSALKEITQCKSLSTLMNILLDLGNAL<br>NGAG<br>LTNQHHRCSVQIARGFKLESLAKLKSTKSFNRRTTALHFLVANIEKCMISGE<br>VSA<br>AAGSQGGGKAKPDAAKHPLEGLFGIMSELPTLQAAKRLVYADSLALIEEM<br>RKGLK<br>VLEAEMTKMSEQGTGESNNQLEEFVDYVKVSISEVENTSQVLQNLQNEAG<br>KMFGH<br>DVSSAESKSKEPAYMFNLLYDFLEGLSSARLERNQVDICHKVNEARTPSPN<br>KTKT<br>ATVGKSDNN*

No.	Sequence ID	Species	Sequence source/database	Amino acid sequence length (aa)	Protein sequence
32	Pt-FH1	*Phaeodactylum tricornutum*	NCBI protein database	2372	MFGDRRSPAARRRAAGNRQAPQAPSHPQTRPPQPVQTFVRSPYDDEASNMSSRRR<br>NLALFKYGSTTKRSTSSMDRSRQHAKEIALKLTTSASLPNSSFGEFADFSSAFGS<br>TATPAPFTTATASRATTTTRSSKKLNDQGRQNQYTSPRTSSQPPVKNEFFDTDPF<br>PDSIGATPAESREEPRRAPPSLPSKEDAFFTAEFGQMSVDTHPNRSTSAQSSSFV<br>VAEWPSEEQSMATMPANSSMQSHYAMSHQEQYQTRPQTPPPPPPRPKQRTQRRVT<br>TPPPNETRTTPRRSVPASLGHEASTTPNQRLVNDPAAVSPNPSVNSSGTAAARRR<br>LRAQRRKGGERTDHGDRQSEPENTLLTTPLSAKRHDNFDNVPVNSGHNATTYAPT<br>TATPPSSRKLQDHTAPLSNRAFSSAALKSTNQSAGSWSTQHRPDLGARQSEPEDA<br>LLTVSVSGTRHSTFDNAPVNNGRSATTYAPTTATPSLSRKLQDHTAPLSNRASSS<br>AALKSTNQSAGSWSTQHRPDLGARQSEPEDALLTMPVSGTRHSNFDNAPVNSGLS<br>ATMYAATTATPPSSRKLQDHTAPLSNRASSSAALKSTNQSAGSWSTQHRPDLGAR<br>QSEPEDPLLTMPVSVKRHSNFENVPVNSGLSATMYAATTATPPSSRKLKDHTAPF<br>SNKVPSSAPGKSTNFSAGSRSMDHRTDHGARPSEPEDAVLTMPVSVKRHSNFDHA<br>PLNSSHSATTHAPITAIPPSSRKLQDHVAHYSNKTSSSAALKSTIHSAGSMSTNR<br>SVSSNTTTKSMGSSTTSSDANVFNSHIESAGFTFDAFGLDASTLNQEVSQAMSEI<br>DTNNVIGFADEFSSSLWMGDSPSSSRSSTPNVSLEEADGFVDGFRVTQPKQNIPI<br>DVKDFPSTTAQSPMNFDTSSSRPFDGHSGHVNRFKGHGEFAVTKRPPLMDEETPL<br>SMKGHSESNATPQPLGNITSATPFRSFGAFTVAASNSKARVKKVTSTSKSGYIQR<br>HPHAHKTVHASPTRMLPATPSQSKPVPTSPRHMQPQNEVEFVDDLESEESTPKAS<br>LVEASEIGARLDIGSESDNGVNSDYIGRPSNLSLLKIPAATASNKDKKKLNPDTL<br>MPEPNETVANEEKKDEDTGASLEKTVGSLKSHWENRVSSAQFTPPACVEGSRRPV<br>IIPREEISGLQSKLGDSILSPERLEQHQSVLSEEAQLPMERITTKAAFSSIQHGL<br>RSSPALDAHETKMTKSEGGAAVRHSHLSQVLNSLQKSEKEDSLRDTQSDTGAPVT<br>FAVRLRKTGFDDRTCSSDDSESRQSGQKKEENETETSPSASDKSTEEIVEHTYGE<br>RCELELQKQREEEDNLQGSKNGEEFSDPYARPQIINRVACNKQKRVDESEEQQSG<br>HQLHSHQLSTTSDECKIEHYTIGVDSSEQSEGSRQDEVPELKSASTTGMLQEKLS<br>PSRRRNSDLIIEPEHDDKSHTSEPKPLVPPSAAVSHLLMLQQLQQRQEPSDH

No.	Sequence ID	Species	Sequence source/database	Amino acid sequence length (aa)	Protein sequence
33	Pt-FH2	*Phaeodactylum tricornutum*	NCBI protein database	2187	MFGDRRSPAARRRAAGNRQAPQAPSHPQTRPPQPVQTFVRSPYDDEASNM<br>SSRRR<br>NLALFKYGSTTKRSTSSMDRSRQHAKEIALKLTTSASLPNSSFGEFADFSSA<br>FGS<br>TATPAPFTTATASRTTTTTRSSKKLNDQGRQNQYTSPRTSSQPPVKNYFFDT<br>DPF<br>PDSIGATPAESREEPRRAPPSLPSKEDAFFTADFGQMSVDTHPKRSTSAQSSS<br>FV<br>VAEWPSEEQSMATMPANSSMQSHYAMSHQEQYQTRPQTPPPPPPRPKQRT<br>QRRVT<br>TPPPNETRTTPRRSVPASLGHEASTTPNQRLVNDPAAVSPNPSVNSSGTAAA<br>RRR<br>LRAQRRKGGERTDHGDRQSEPENTLLTTPLSAKRHDNFDNVPVNSGHNAT<br>TYAPT<br>TATPPSSRKLQDHTAPLSNRAFSSAALKSTNQSAGSWSTQHRPDLGARQSE<br>PEDA<br>LLTVSVSGNRHSNFDNAPVNSGLSAMMYAAITATPPSSRKLQDHTAPLSNR<br>ASSS<br>AALKSTNQSAGSWSTQHRPDRGARQSEPEDAVLTMPVSVKRHSNFDHAPL<br>NSSHS<br>ATTHAPITAIPPSSRKLQDHVAHYSNKTSSSAALKSTIHSAGSMSTNRSVSS<br>NTT<br>TKSMGSSTTSSDANVFNSHIESAGFTFDAFGLDASTLNQEVSQAMSEIDTN<br>NVIG<br>FADEFSSSLWMGDSPSSSRSSTPNVSLEEADGFVDGFRVTQPKQNIPIDVKD<br>FPS<br>TTAQSPMNFDTSSSRPFDGHSGHVNRFKGHGEFAVTKRPPLMDEETPLSM<br>KGHSE<br>SNATPQPLGNITSATPFRSFGAFTVAASNSKARVKKVTSTSKSGYIQRHPHA<br>HKT<br>VHASPTRMLPATPSQSKPVPTSPRHMQPQNEVEFVDDLESEESTPKASLVE<br>ASEI<br>GARLDIGSESDNGVNSDYIGRPSNLSLLKIPAATASNKDKKKLNPDTLMPEP<br>NET<br>VANEEKKDEDTGASLEKTVGSLKSHWENRVSSAQFTPPACVEGSRRPVLIP<br>REEI<br>SGLQSKLGDSILSPERLEQHQSVMSEEAQLPMERITTKAAFSSIQQGLRSSPV<br>LD<br>AHETKMTKSEGGAAVRHSHLSQVLNSLQKSEKEDSLRDTQSDTGAPVTFA<br>VRLRK<br>TVFDDRTCSSDDSESRQSGQKKEENETETSPSASDKSTEEIVKHTYGERCEL<br>DLQ<br>KQREEEDNLQGSKNGEEFSDPYARPQIINRVACNKQKRVDESEEQQSGHQL<br>HSHQ<br>LSTTSDECKIEHYTIGVDSSEQSEGSRQDEVPELKSASTTGMLQEKLSPSRR<br>RNS<br>DLIIEPEHDDKSHTSEPKPLVPPSAAVSHLLMLQQLQQRQEPSDHNSVASGR<br>GSL<br>SVMTTDTVVSEESQPRSTKAHTPKATMMMLNAFLAGRESITSCSEDKQAK<br>PDLID<br>FEGEGSDFKSPPKGAQPAPNSNLPALKDDPTYERYFKMLKVSMPLEVVKH<br>AMIRD<br>GLDPTVLDGDHNKPAGILLKDDPAYQKYFKMLNIGLPMEAVKHAMERDG

No.	Sequence ID	Species	Sequence source/database	Amino acid sequence length (aa)	Protein sequence
34	Pt-FH3	*Phaeodactylum tricornutum*	NCBI protein database	2016	MTSSLGESALRVPQTSPTQIPPKKTSSTSNPPLHPVLKSCRKRSVPAQLDLH<br>YIT<br>DHILVTSAPVEALQPTYKDIGRNPQPPNRTSSAEQTELRNTSAALSHAETPL<br>SDS<br>EPKDSAIDPAQTSSQRKPVANHAGSPSTSLHGPQVSSIAAGNSAVDLSLFLE<br>TQF<br>ASANYLLVCLSDNLPDDHFLILLRRQIVRLPWTAPGSPASETPTVSTLLRFC<br>YLL<br>HAWLHDPEHVVVVYCENGKTRSGLAVAAYLAFAGKSGVETCAQGFAHF<br>VSCRCPE<br>APDPRAAFSSLPASLRLLFRNFDQGIEAGLKRNREPLLLRAISLQGVPVEEK<br>PCL<br>DIWDAQGRHIYSSHPDLWDDLLETFPVLAGNDCNNLSSRASQWADEEGFF<br>KVNVV<br>LEGDFCLLCRFGGAHANKGLGETSTQLFRYVNTTVAMSTGQCELRFMHV<br>DVMRRY<br>VPFLNSDDFLLTLVLEADWNCRRKAMVPLLLNSCNAEAALDFLKGDKARE<br>KGWQV<br>LSAYHAAQPSPFDSACFVETLETSLQSCPSNLIALALQLSNFDYKAALNML<br>MKRS<br>PCPWRQGLNWGDDPTAHADAPTALVQDTVYSLDTASQGSATDSSLSGFTR<br>CSGEP<br>SRREDRANNGPPLVPSKTSRVGPISPIPLEALENLEATDFTSEMSSGAIVPQQ<br>ML<br>DLNEEAIFSSRLSRSSSSDNKPDALSNTQIGKLSNPKSEVSMELAIKADPRFE<br>KY<br>YKMHAVGLPEGAIRNACVRDGVDASFLDLDWDRSYESQSKASPESSGDTD<br>PPMKS<br>DPRFEKYYKMHAVGLPEGAIRNACVRDGVDASFLDLDWDRSYESQSKASP<br>KSSGD<br>TDPPMKSDPRFEKYYKMHTVGLPEGAIRNACVRDGVDASFLDLDWDRSY<br>ESQSKA<br>SPESSGDTDPPMKSDPRFEKYYKMHAVGLPEGAIRNACVRDGVDASFLDL<br>DWDRS<br>YESQSKASPKSSGDTDLPMKSDPRFEKYYKMHAVGLPEGAIRNACVRDGV<br>DASFL<br>DLDWDRSYESQSKASPKSSGDTDPPMKSDPRFEKYYKMHAVGLPEGAIRN<br>ACVRD<br>GVDASFLDLDWDRSYESQSKASPKSSGDTDLPMKSDPRFEKYYKMHAVG<br>LPEGAI<br>RNACVRDGVDASFLDLDWDRSYESQSKASPKSSGDTDPPMKSDPRFEKYY<br>KMHAV<br>GLPEGAIRNACVRDGVDASFLDLDWGRSYISQTTKPINDDMNSGTPVKDD<br>LEYAK<br>YFKMLHMGLPPGAVENALTRDGKDPKILKFVQNKSVDFQLTSGGRHTNSA<br>TKEAS<br>RVRRKKIYWNTLDHSRIKEDSLWTLVRNRVHMSHLNYDVKEFKELFTESA<br>DPAQQ<br>RRKKQQRDTTKAKKSVQVIDGKRSMNGGIILLRLKLDYKKIARLVNSMEH<br>GKLDA<br>TQIMALKEFLPSIEERRGLSTYMKTHGQSDETKMKAYHELSECEKYMFTM<br>MEVSD<br>APQKFDCMLFRVQFKIRFAEVMESVRVVQRACEEVRSSERLREILAIILTLV

No.	Sequence ID	Species	Sequence source/database	Amino acid sequence length (aa)	Protein sequence
35	Pt-FH4	*Phaeodactylum tricornutum*	NCBI protein database	1002	MRLYNANTCTPAVTSSGTRSCFQRSEPRIQSLAVKVNNKPPPTTTQLDTQS<br>LKLE<br>APTQRKVKEKFQSPIPSSHDEYHITRNPPHFLSRNTTADTSVSSGTFCGIFNR<br>NS<br>SRSFHFDVDGATCNDLDERCRGASSPFPSDFSASTLTTVSAFETYQDLLEKG<br>HPP<br>YQPIESAVSSSVEKSCLGQELIWPSPCRVRPRPRLSDVEPKDFGIEEIENVAA<br>RA<br>KPNPDHKTPSQWKLHENCAFLPRNLAASVPEPKRTQSKKWNTFGASRRSN<br>VDANN<br>DMPDPQCLTPLTWVPFVTRFESSPASIEDSISTLFEPIEFPKDAPSKSMIVASP<br>K<br>PKGYFSKDRGRGVSGRDSFKGHRQKLHASDADSQTLEKAEDGILSRERIGG<br>KVFQ<br>RIPGLSKCAKMTTPQIYSNRKYIAASSGYSETTGTFSVLDEQLHSPDLGAAS<br>LEQ<br>IFQKERLRKFQTNGKEMKQFKGKVDVEQKHSEIVVNLENQPTNYAHKSAF<br>SATKK<br>AVKAVPSSDDLEKANMLDLTNKGPSDKMIPDLPEKYTKMLKVGLSRAATE<br>NSMRR<br>DGIDPDDFRPPETTSSKLLPTVAAKDPFRRFRIHWEAHTNVRSNTVWAMIG<br>RDDQ<br>WLLNLAIDHDEMFTLFSETRDSAPHTRKPGQRKNNANAVKVIDSKRAKNG<br>GIILA<br>RIRMSYKEIVRAIDSMDETALTLEQIKGIGFLVPTLEEREALKRHIERSGEEL<br>TT<br>ECEKFMSEIISVEDPARNLEAMLFMKKFPFCVLELKNGKKDEVKGHRWQP<br>RLLNV<br>SSRFTDAKTIQKVCDEILGASKLRRVLGVILRLGNELNQADVAAPKGAVDA<br>VTVK<br>SLSKLNQDKAFDQKMTFLQYAAQIIRENNEELLLFKDDMPSLKLAEKISWE<br>ESMQ<br>NVESIENSLTRLRKIALGRAEDDSAEKERGELPTENEFRVLQRTSIGRFIFNS<br>YV<br>EINSMYKEIEAAKSAFESLLNYFGEEATEPEVQHFLHFLFEFCNDFENAAER<br>AVD<br>KGNHYAKSERR*

No.	Sequence ID	Species	Sequence source/database	Amino acid sequence length (aa)	Protein sequence
36	Pt-FH5	*Phaeodactylum tricornutum*	NCBI protein database	2133	MSYRQRIPMDPDGTNEAVNLAKNYLDPPDNEPSFSLNARQLGLLKSRQQR<br>DPSVI<br>NEKQPFVSRSNMSQQQHKPKSDNDFGTSAVKPRAHGPMRTPNPISRFSAA<br>AAHRR<br>QPLADEQGRAAYLRPLLQQRALKKSSTTSADSDPSGKALLRRMLAKVVVK<br>KSKST<br>STGASSENNPDRGLPSKTMMDKDRAKTTETNRSERQEAREACKYTSPVDN<br>TVAPK<br>RSRQVDEGLKYRIHEKLRQKPFKMHPCDLKVSAIERPLVMSDDDDGLEGY<br>EHFIK<br>QLRGEVSGKHWQLWRSFSALMTGGDPPDEGIMHIDRPCEFDERTRKNLSV<br>LNLHL<br>KKYGEAECNSKTQLINDRRLSKERINRSYPANQRQAVSNIESSASSSSENSA<br>LEN<br>DAVLQMRSSLSTALEFSQIELSESLRKYHTDKGASGSSNQQHDDLESAKYH<br>TAKE<br>KDNCAGAKEAPSETVTGSENRASNSSIVSLDQFELRPSPPSDANGSIIWSDV<br>RLR<br>SLDQVSYNSELDSIKASTPVWANMKLRPVVNFSKLGDDVRATNSADFDSIP<br>QVNS<br>GTSQKPIDTEDFDKLQSTDTGTEKKPIFVQTPTAVVYKQPLMDGIERCEKPR<br>PEQ<br>RQLLQPTPIPHSAQVLTGPKPEDAHPRVTKLSTGPIGQASGDKIDEATVFKV<br>KQT<br>TNSSLEVERRVIVGKKMIMLVESPVGDSRASVLWKLAREKVESLTLDMAL<br>QKVAL<br>LCFKGSSRKVLDFISSEDCLKFANTFYNMNAVVPLSKTDPMVSASLQSDDA<br>EIDD<br>SSTTDRVASLNDEEQTLLEMFRLLRRTQPAENAFDTSLSRRGSEAKTLSSTL<br>TPK<br>EEEIAKKYLMMLELKVPLNEVKSSLLKDGVDSKIVTKIMREAVDMPVNVV<br>NAPDL<br>PSSPVSTMTCSVAQVSDESEIAAPYKKMIRLRIPAEAVRHKMIKDEISPRIID<br>LV<br>LGTGGAVYNKINDDLALSSEEAVLVLKYQKMLKISIAPEAVRHKMRQDQV<br>NNKII<br>AAVFSQNEKEKVEAPRLNLSIEEEKIAEKYREMVRLKVPKEGVRHRMTKD<br>QIDKK<br>IVDSVLQDDSCTSKDTSANLPDHRKVPSSSLTSDEEAVVLQYKRLLKLQIPK<br>AAI<br>LDRMEKEGASANVIKAVLGNVTSKCNPGEKDTDSQSKTGTKLVSLHWTPL<br>SGTAL<br>DNSVWAARKRRKDELPQPEGSDIFQLIHLFQKKTANTSVKEGSVDESSSAQ<br>GKAM<br>LLEVTRSNNVAISLKAFKDFSFLELAETIALLDPLRKIRGDRILFLRDLLPTIS<br>E<br>VQTILAYKGTDDRLVPAERWFRATAQIRRIETKVQVLQSMETLRSEILALC<br>ENFR<br>LLARACRQVMESEKLEALLEMVLRVGNIMNEGTRTGGAAGFKFDSLLRLT<br>QTKSS<br>DGRTTVLDFLVTVFVAKGSRGTLNLSSDISDCHMASRMLMSDMSNDVKNI<br>RQALD<br>VCSTELAALRDEITSSTIEGKKSGQCSDLKRTEHAVFSKREEFLAAVSKSSE

No.	Sequence ID	Species	Sequence source/database	Amino acid sequence length (aa)	Protein sequence
37	Sc-FH1	*Saccharomyces cerevisiae*	Saccharomyces Genome Database (SGD)	1954	MLKNSGSKHSNSKESHSNSSSGIFQNLKRLANSNATNSNTGSPTYASQQHSPVG<br>NEVSTSPASSSSFRKLNAPSRSTSTEARPLNKKSTLNTQNLSQYMNGKLSGDVPV<br>SSQHARSHSMQSKYSYSKRNSSQASNKLTRQHTGQSHSASSLLSQGSLTNLSKFT<br>TPDGKIYLEMPSDPYEVEVLFEDIMYKRNIFQSLSEDKQEALMGYSIEKKWLIVK<br>QDLQNELKKMRANTTSSSTASRTSMASDHHPILTANSSLSSPKSVLMTSASSPTS<br>TVYSNSLNHSTTLSSVGTSTSKGKKLVSGSLKKQPSLNNIYRGGAENNTSASTLP<br>GDRTNRPPIHYVQRILADKLTSDEMKDLWVTLRTEQLDWVDAFIDHQGHIAMANV<br>LMNSIYKTAPRENLTKELLEKENSFFKCFRVLSMLSQGLYEFSTHRLMTDTVAEG<br>LFSTKLATRKMATEIFVCMLEKKNKSRFEAVLTSLDKKFRIGQNLHMIQNFKKMP<br>QYFSHLTLESHLKIIQAWLFAVEQTLDGRGKMGSLVGASDEFKNGGGENAILEYC<br>QWTMVFINHLCSCSDNINQRMLLRTKLENCGILRIMNKIKLLDYDKVIDQIELYD<br>NNKLDDFNVKLEANNKAFNVDLHDPLSLLKNLWDICKGTENEKLLVSLVQHLFLS<br>SSKLIEENQNSSKLTKQLKLMDSLVTNVSVASTSDEETNMNMAIQRLYDAMQTDE<br>VARRAILESRALTKKLEEIQAERDSLSEKLSKAEHGLVGQLEDELHERDRILAKN<br>QRVMQQLEAELEELKKKHLLEKHQQEVELRKMLTILNSRPEESFNKNEGTRGMNS<br>SLNSSEKANIQKVLQDGLSRAKKDYKDDSKKFGMTLQPNKRLKMLRMQMENIENE<br>ARQLEMTNFAEFEKDRLEPPIHIKKPKVKKMKNKDRKPLVKPQEADVNKLNDLRR<br>ALAEIQMESNDISKFNVEERVNELFNEKKSLALKRLKELETKYKGFGIDFNVDEI<br>MDSPKKNTGDVETEEDANYASLDPKTYQKKLDEINRITDQLLDIQTQTEHEIQVE<br>EDGESDLSSSSSDDESEEIYQDASPTQELRSEHSELSSGSGPGSFLDALSQKYGT<br>GQNVTASAAFGENNNGSGIGPLHSKVEKTFMNRLRKSTVSSAPYLEELTQKVNKV<br>EPYEQNEDEGLDKKSLPENSTASAASAFDKAEKDMRQHVENGKQGRVVNHEEDKT<br>ADFSAVSKLNNTDGAEDLSTQSSVLSSQPPPPPPPPPPVPAKLFGESLEKEKKSE<br>DDTVKQETTGDSPAPPPPPPPPPPPPMALFGKPKGETPPPPPLPSVLSSSTDGVI<br>PPAPPMMPASQIKSAVTSPLLPQSPSLFEKYPRPHKKLKQLHWEKLDCTDNSIWG<br>TGKAEKFADDLYEKGVLADLEKAFAAREIKSLASKRKEDLQKITFLSRDISQQFG<br>INLHMYSSLSVADLVKKILNCDRDFLQTPSVVEFLSKSEIIEVSVNLARNYA

No.	Sequence ID	Species	Sequence source/database	Amino acid sequence length (aa)	Protein sequence
38	Sc-FH2	*Saccharomyces cerevisiae*	Saccharomyces Genome Database (SGD)	1376	MDSSPNKKTYRYPRRSLSLHARDRVSEARKLEELNLNDGLVAAGLQLVGV<br>ALEKQ<br>GTGSHIYMKQKNFSANDVSSSPMVSEEVNGSEMDFNPKCMPQDASLVER<br>MFDELL<br>KDGTFFWGAAYKNLQNISLRRKWLLICKIRSSNHWGKKKVTSSTTYSTHL<br>ATNEL<br>AENAHFLDGLVRNLSTGGMKLSKALYKLEKFLRKQSFLQLFLKDEIYLTTL<br>IEKT<br>LPLISKELQFVYLRCFKILMNNPLARIRALHSEPLIRWFTELLTDQNSNLKCQ<br>LL<br>SMELLLLLTYVEGSTGCELIWDQLSILFTDWLEWFDKILADDIAIHSSLYLN<br>WNQ<br>LKIDYSTTFLLLINSILQGFNNKTALEILNFLKKNNIHNTITFLELAYKDDPNS<br>V<br>VIMEQIKQFKSKESAIFDSMIKTTNDTNSLHPTKDIARIESEPLCLENCLLLK<br>AK<br>DSPVEAPINEIIQSLWKILDSQKPYSESIKLLKLINSLLFYLIDSFQVSTNPSFD<br>ETLESAENVDYVFQDSVNKLLDSLQSDEIARRAVTEIDDLNAKISHLNEKL<br>NLVE<br>NHDKDHLIAKLDESESLISLKTKEIENLKLQLKATKKRLDQITTHQRLYDQP<br>PSL<br>ASSNLSIAGSIIKNNSHGNIIFQNLAKKQQQQKISLPKRSTSLLKSKRVTSLS<br>S<br>YLTDANNENESQNESEDKSKDSLFQRSTSTINFNIPSMKNITNMQNVSLNSI<br>LSE<br>LEFSNSLGTQPNYQSSPVLSSVSSSPKLFPRLSSDSLDNGIQLVPEVVKLPQL<br>PP<br>PPPPPPPPPLPQSLLTEAEAKPDGVSCIAAPAPPPLPDLFKTKTCGAVPPPPPP<br>P<br>PLPESLSMNKGPSNHDLVTPPAPPLPNGLLSSSSVSINPTTTDLKPPPTEKRL<br>KQ<br>IHWDKVEDIKDTLWEDTFQRQETIKELQTDGIFSQIEDIFKMKSPTKIANKR<br>NAE<br>SSIALSSNNGKSSNELKKISFLSRDLAQQFGINLHMFSQLSDMEFVMKVLNC<br>DND<br>IVQNVNILKFFCKEELVNIPKSMLNKYEPYSQGKDGKAVSDLQRADRIFLE<br>LCIN<br>LRFYWNARSKSLLTLSTYERDYYDLIFKLQKIDDAISHLNRSPKFKSLMFIIT<br>EI<br>GNHMNKRIVKGIKLKSLTKLAFVRSSIDQNVSFLHFIEKVIRIKYPDIYGFVD<br>DL<br>KNIEDLGKISLEHVESECHEFHKKIEDLVTQFQVGKLSKEENLDPRDQIIKK<br>VKF<br>KINRAKTKSELLIGQCKLTLIDLNKLMKYYGEDPKDKESKNEFFQPFIEFLA<br>MFK<br>KCAKENIEKEEMERVYEQRKSLLDMRTSSNKKSNGSDENDGEKVNRDAV<br>DLLISK<br>LREVKKDPEPLRRRKSTKLNEIAINVHEGDVKTRKDEDHVLLERTHAMLN<br>DIQNI<br>*
