# Supplementary material for: Identification and Expression Analysis of the Formin Gene Family in Neopyropia yezoensis Under Different Environmental Conditions
Source: Genes (Basel). 2026 Sep 10;17(9):1089. doi: 10.3390/genes17091089 (PMC13606620; doi:10.3390/genes17091089)
Supplement: Supplementary file 1 [file genes-17-01089-s001.zip › Supplementary Table S2.pdf]

Supplementary Table S2. qRT-PCR amplification program

Step	Temperature	Time	Number of cycles	Fluorescence acquisition
Initial denaturation	95 ℃	30 s	1	
Denaturation	95 ℃	10 s	40	
Annealing/extension	60 ℃	30 s	40	Yes
Melting-curve analysis	60–95 ℃	0.3 ℃ increments		Yes
