# Supplementary material for: Identification and Expression Analysis of the Formin Gene Family in Neopyropia yezoensis Under Different Environmental Conditions
Source: Genes (Basel). 2026 Sep 10;17(9):1089. doi: 10.3390/genes17091089 (PMC13606620; doi:10.3390/genes17091089)
Supplement: Supplementary file 1 [file genes-17-01089-s001.zip › Supplementary Table S3.pdf]

Supplementary Table S3. Amplification efficiencies and correlation coefficients (R^2) of qRT-PCR primers determined by standard curve analysis

Gene name	Amplified fragment size (bp)	Efficiency (%)	R^2
NpyFormin01	197	102	0.999
NpyFormin02	262	95	0.9996
NpyFormin03	295	92	0.9996
NpyUBC	112	99[a]	-
NpyMAP	200	95[a]	-

a The amplification efficiencies of the NpyUBC and NpyMAP reference gene primers were obtained from Gao et al. (2018) .
