# Supplementary material for: Identification and Expression Analysis of the Formin Gene Family in Neopyropia yezoensis Under Different Environmental Conditions
Source: Genes (Basel). 2026 Sep 10;17(9):1089. doi: 10.3390/genes17091089 (PMC13606620; doi:10.3390/genes17091089)
Supplement: Supplementary file 1 [file genes-17-01089-s001.zip › Supplementary Table S4.pdf]

Supplementary Table S4. Ct values of NpyUBC and NpyMAP under different experimental treatments

Treatment	NpyMAP (Ct, Mean ± SD)	NpyUBC (Ct, Mean ± SD)
Drought control	27.21 ± 0.39	23.06 ± 0.60
30% dehydration	26.09 ± 0.30	22.15 ± 0.22
80% dehydration	27.38 ± 0.48	22.59 ± 0.30
Rehydration	26.53 ± 0.72	22.32 ± 0.25
Temperature control	26.15 ± 0.42	22.32 ± 0.60
Low temperature	26.81 ± 1.28	22.03 ± 0.58
High temperature	27.01 ± 1.37	22.04 ± 0.71
Light control	26.15 ± 0.42	22.32 ± 0.60
Low light	27.83 ± 0.61	22.42 ± 0.78
High light	26.15 ± 0.81	21.25 ± 1.18

Ct values represent the mean ± standard deviation (SD) calculated from samples under each treatment condition.
