# Supplementary material for: Identification and Expression Analysis of the Formin Gene Family in Neopyropia yezoensis Under Different Environmental Conditions
Source: Genes (Basel). 2026 Sep 10;17(9):1089. doi: 10.3390/genes17091089 (PMC13606620; doi:10.3390/genes17091089)
Supplement: Supplementary file 1 [file genes-17-01089-s001.zip › Supplementary Table S5.pdf]

Supplementary Table S5. P2Rank-predicted ligand-binding pockets and corresponding residues of NpyFormin02

Structure	Pocket	Score	Probability	No. of Residues	Residue IDs
NpyFormin02	Pocket 1	13.18	0.697	14	A_457, A_458, A_461, A_464, A_465, A_470, A_473, A_476, A_477, A_480, A_483, A_484, A_487, A_495
NpyFormin02	Pocket 2	7.33	0.423	10	A_261, A_263, A_275, A_276, A_277, A_278, A_283, A_288, A_291, A_293
NpyFormin02	Pocket 3	3.66	0.144	13	A_210, A_211, A_214, A_218, A_369, A_418, A_419, A_422, A_426, A_533, A_536, A_537, A_540
NpyFormin02	Pocket 4	2.96	0.097	11	A_214, A_218, A_369, A_372, A_415, A_418, A_419, A_422, A_426, A_540, A_543
NpyFormin02	Pocket 5	1.72	0.029	8	A_338, A_341, A_346, A_347, A_348, A_396, A_397, A_400
