# Supplementary material for: Identification and Expression Analysis of the Formin Gene Family in Neopyropia yezoensis Under Different Environmental Conditions
Source: Genes (Basel). 2026 Sep 10;17(9):1089. doi: 10.3390/genes17091089 (PMC13606620; doi:10.3390/genes17091089)
Supplement: Supplementary file 1 [file genes-17-01089-s001.zip › Supplementary Table S6.pdf]

Supplementary Table S6. P2Rank-predicted ligand-binding pockets and corresponding residues of NpyFormin03

Structure	Pocket	Score	Probability	No. of Residues	Residue IDs
NpyFormin03	Pocket 1	14.91	0.746	14	A_428, A_432, A_435, A_436, A_441, A_444, A_446, A_447, A_448, A_451, A_454, A_455, A_458, A_466
NpyFormin03	Pocket 2	8.2	0.482	11	A_415, A_421, A_422, A_425, A_484, A_487, A_566, A_567, A_585, A_588, A_589
NpyFormin03	Pocket 3	4.13	0.178	13	A_38, A_395, A_398, A_399, A_402, A_41, A_46, A_47, A_538, A_541, A_544, A_545, A_548
NpyFormin03	Pocket 4	2.77	0.085	11	A_331, A_332, A_391, A_392, A_44, A_45, A_46, A_534, A_535, A_538, A_541
NpyFormin03	Pocket 5	1.91	0.038	9	A_422, A_425, A_429, A_445, A_446, A_447, A_449, A_566, A_567
